# The Secular Trends in the Incidence Rate and Outcomes of Out-of-Hospital Cardiac Arrest in Taiwan—A Nationwide Population-Based Study

**DOI:** 10.1371/journal.pone.0122675

**Published:** 2015-04-15

**Authors:** Cheng-Yi Wang, Jen-Yu Wang, Nai-Chi Teng, Ting-Ting Chao, Shu-Ling Tsai, Chi-Liang Chen, Jeng-Yuan Hsu, Chin-Pyng Wu, Chih-Cheng Lai, Likwang Chen

**Affiliations:** 1 Department of Internal Medicine, Cardinal Tien Hospital, Fu Jen Catholic University College of Medicine, New Taipei City, Taiwan; 2 Institute of Population Health Sciences, National Health Research Institutes, Zhunan Town, Miaoli County, Taiwan; 3 Medical Research Center, Cardinal Tien Hospital, Fu Jen Catholic University College of Medicine, New Taipei City, Taiwan; 4 National Health Insurance Administration, Ministry of Health and Welfare, Taipei City, Taiwan; 5 The Department of Accounting, The College of Business, Chung Yuan Christian University, Chung Li District, Taoyuan City, Taiwan; 6 Department of Internal Medicine, Taichung Veterans General Hospital, Taichung City, Taiwan; 7 Departments of Internal Medicine, Landseed Hospital, Ping Jen District, Taoyuan City, Taiwan; 8 Department of Intensive Care Medicine, Chi Mei Medical Center, Liouying, Tainan City, Taiwan; San Raffaele Scientific Institute, ITALY

## Abstract

**Objective:**

This study investigated the trends in incidence and mortality of out-of-hospital cardiac arrest (OHCA), as well as factors associated with OHCA outcomes in Taiwan.

**Methods:**

Our study included OHCA patients requiring cardiopulmonary resuscitation (CPR) upon arrival at the hospital. We used national time-series data on annual OHCA incidence rates and mortality rates from 2000 to 2012, and individual demographic and clinical data for all OHCA patients requiring mechanical ventilation (MV) care from March of 2010 to September of 2011. Analytic techniques included the time-series regression and the logistic regression.

**Results:**

There were 117,787 OHCAs in total. The overall incidence rate during the 13 years was 51.1 per 100,000 persons, and the secular trend indicates a sharp increase in the early 2000s and a decrease afterwards. The trend in mortality was also curvilinear, revealing a substantial increase in the early 2000s, a subsequent steep decline and finally a modest increase. Both the 30-day and 180-day mortality rates had a long-term decreasing trend over the period (p<0.01). For both incidence and mortality rates, a significant second-order autoregressive effect emerged. Among OHCA patients with MV, 1-day, 30-day and 180-day mortality rates were 31.3%, 75.8%, and 86.0%, respectively. In this cohort, older age, the female gender, and a Charlson comorbidity index score ≥ 2 were associated with higher 180-day mortality; patients delivered to regional hospitals and those residing in non-metropolitan areas had higher death risk.

**Conclusions:**

Overall, both the 30-day and the 180-day mortality rates after OHCA had a long-term decreasing trend, while the 1-day mortality had no long-term decline. Among OHCA patients requiring MV, those delivered to regional hospitals and those residing in non-metropolitan areas tended to have higher mortality, suggesting a need for effort to further standardize and improve in-hospital care across hospitals and to advance pre-hospital care in non-metropolitan areas.

## Introduction

Out-of-hospital cardiac arrests (OHCAs) remain a major event which affects public health worldwide. The incidence of OHCA varies from 37 to 121 per population of 100,000 people, and the rate of survival to hospital discharge ranges from 4.5% to 10.7% [1–8]. The variation may be due to different definitions, outcome measures, study sites, and populations. In general, western countries have more studies on this topic [1–8], while some Asian countries have also paid much attention to this problem in recent years [9, 10].

There has been little literature on OHCA in Taiwan. A recent article reported results from studying a random sample of Taiwanese OHCA patients who were 15 years of age or older, had non-traumatic conditions and received intubation in years from 2005 to 2007 [11]. The results indicate a 13% probability of survival to hospital admission, and a 1-month survival rate of 17.3% among admitted OHCA patients. Another recent study investigated OHCA survival rates among Taiwanese adult OHCA patients in a nonmetropolitan area in 2008 [12]. Its findings show that the probability of survival to admission was 16.3%, and the chance of surviving to discharge was 1.4% among these OHCA patients.

There is still a lack of literature on OHCA in Taiwan, particularly on long-term trends in incidence rates and mortality rates, and factors associated with long-term prognosis. Advances in knowledge on the secular trends can help develop cardiovascular disease preventive strategies. Further information on factors associated with post-OHCA prognosis can facilitate improvement in pre-hospital and in-hospital care to reduce harms from OHCA, as well as physician-patient communication regarding post-OHCA care planning. Our study intends to furnish knowledge on OHCA in Asia. It examined OHCA incidence rates and mortality rates in Taiwanese adults from the early 2000s to the early 2010s, and explored the patterns of time trends, as well as differences between genders and among age groups. It also investigated factors associated with short-term, mid-term and long-term mortality rates among OHCA patients requiring mechanical ventilation (MV) care during the hospital stay.

## Materials and Methods

### Study setting

We used two databases constructed by the National Health Insurance Administration (NHIA) and National Health Research Institutes (NHRI) of Taiwan. One has time-series data on annual OHCA incidence rates and mortality rates in Taiwan from 2000 to 2012. The other has data on individual demographic and clinical characteristics for all OHCA patients using MV care during the period from March of 2010 to September of 2011. The records and information of patient were de-identified prior to analysis. Therefore, informed consent was waived by regulations in Taiwan. Ethics approval was obtained from Institution Review Board of Cardinal Tien Hospital.

The Taiwan National Health Insurance (NHI) covers nearly all residents of Taiwan, and has contracted with over 90% of all healthcare organizations in Taiwan [13]. As shown in S1 Table, in year 2000, 94% of hospitals in Taiwan provided NHI care; this rate increased to 98% in 2012. The Taiwan National Health Interview Survey data show that the NHI provides services in almost each outpatient visit and each hospital stay [14]. The NHIA has been the single buyer since the launch of NHI [15], and thus has great influences on behaviors among care providers, as well as care consumers. It had constructed a scheme of constantly and regularly building NHI databases before the establishment of NHI, and the Taiwan government uses the data mainly for administrative purposes and partly for research helpful for improving the NHI and the national health.

The NHI databases contain rich information on individual disease diagnosis, healthcare use, and times of using care in healthcare organizations, in addition to the features of healthcare organizations providing care and individual socioeconomic background. The NHI database within the NHIA also contains information on the time of death for deceased patients, which makes it possible to study the length of survival time after OHCA. Therefore, this data source is suitable for performing a national population-base study to precisely estimate the incidence rate and the mortality rates of OHCA, and to investigate factors associated with OHCA outcomes.

### Identification of OHCA patients and their clinical characteristics

**Fig 1:**
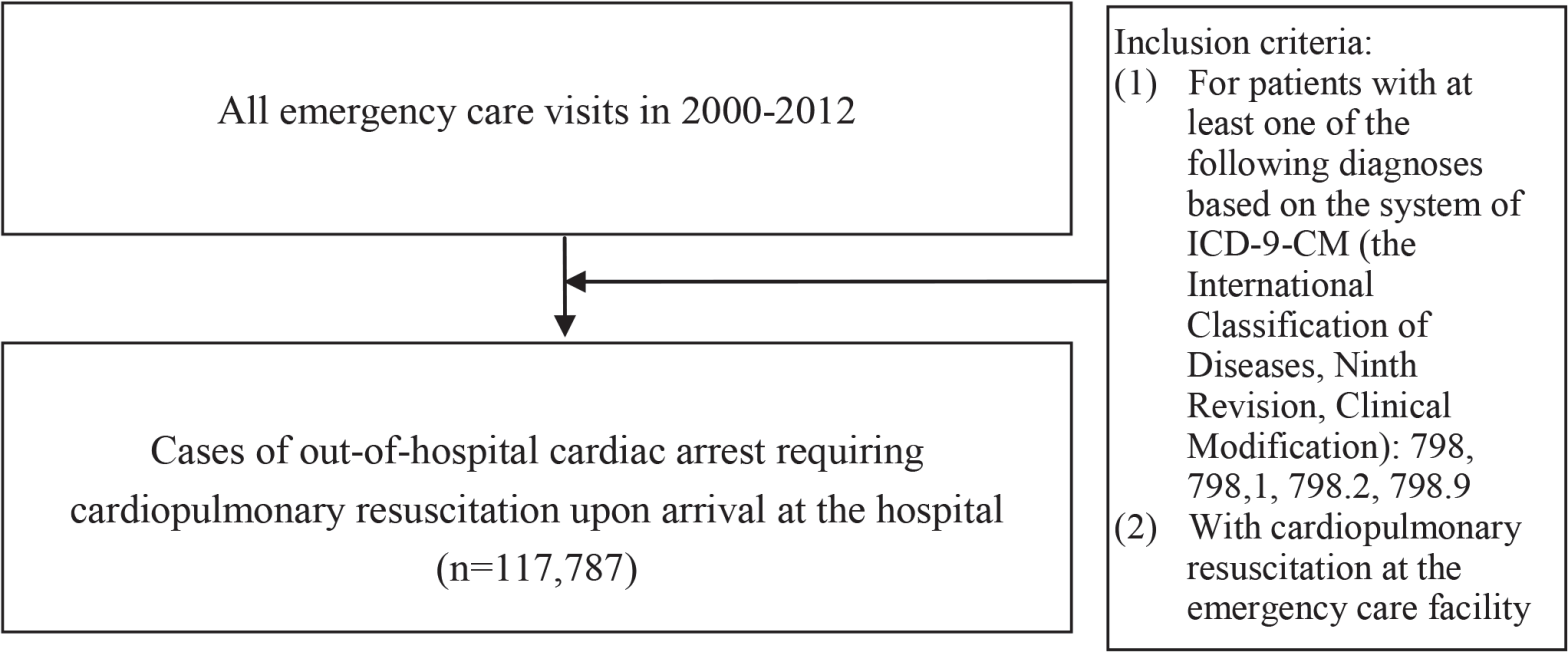
The selection process of study subjects with out-of-hospital cardiac arrest requiring cardiopulmonary resuscitation upon arrival.

**Fig 2:**
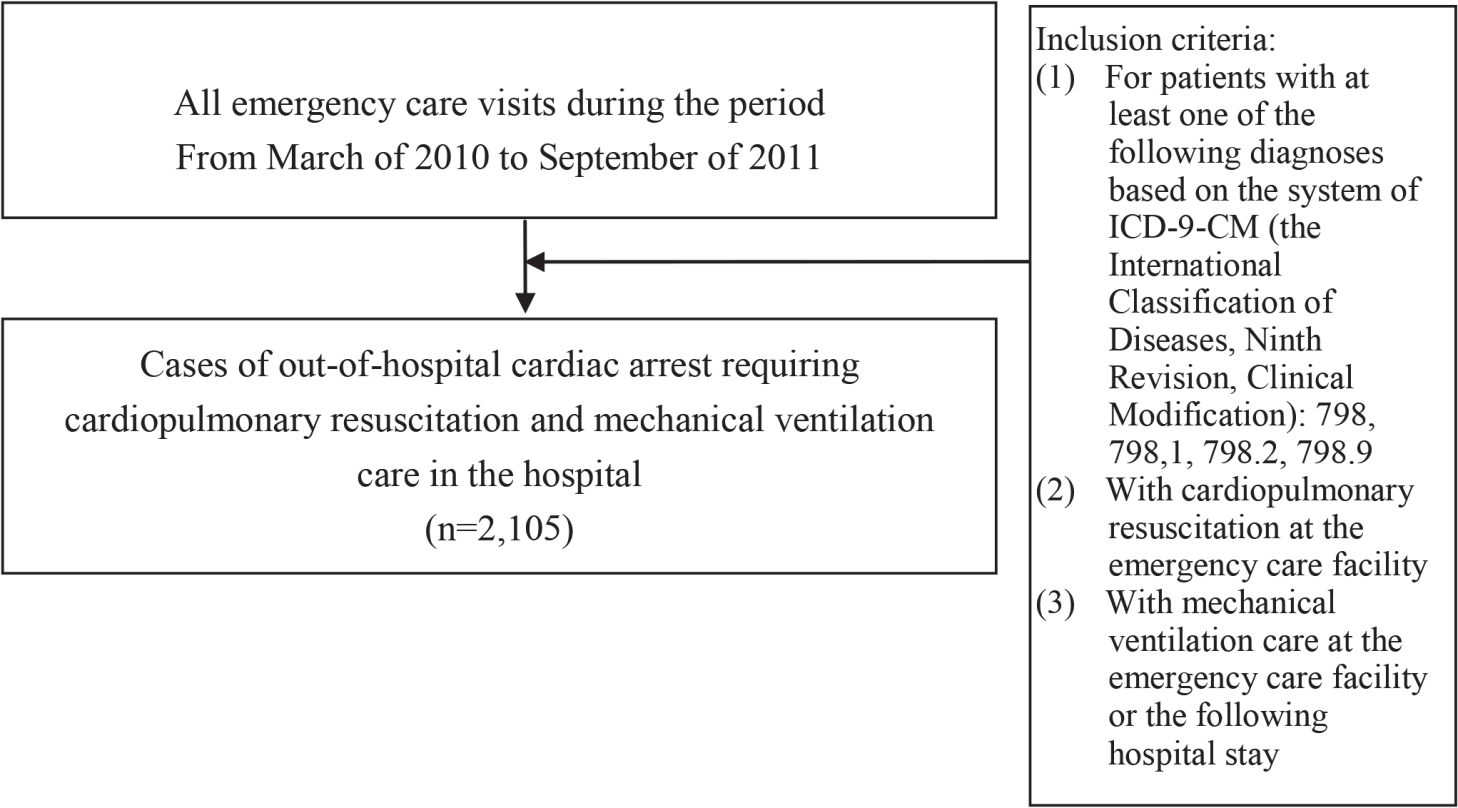
Selection of study subjects with out-of-hospital cardiac arrest requiring cardiopulmonary resuscitation and mechanical ventilation.

We identified all patients who were sent to emergency care (ER) facilities with diagnosis of OHCA and receipt of CPR upon arrival between January 1, 2000, and December 31, 2012 (Fig 1). The OHCA diagnosis codes are 798, 798.1, 798.2, and 798.9 according to the International Classification of Diseases, Ninth Revision, Clinical Modification (ICD-9-CM). Our study focuses on OHCA requiring CPR upon arrival at the hospital. Furthermore, we also investigated a cohort of OHCA patients requiring CPR upon arrival at the hospital and using MV care during the hospital stay (Fig 2). For this OHCA cohort using MV, we further identified their conditions regarding organ dysfunctions and diabetes. S2 Table lists operational definitions regarding these clinical features. We examined their longitudinal data on diagnosis and use of healthcare and medication, as well as their status in an NHI catastrophic illness registry system that the NHIA has been operating for 20 years in order to help persons with severe diseases and heavy financial burden from such diseases.

The NHI data have been broadly used in research on clinical epidemiology and health services as well as other health-related fields in Taiwan, and the quality of data has been recognized [16]. Over recent years, there have also been more and more studies on validation of specific disease diagnoses in the NHI data. Examples include diagnoses of atrial fibrillation, acute myocardial infarction, congestive heart failure, chronic kidney disease, chronic obstructive pulmonary disease, diabetes mellitus, gastrointestinal bleeding, gout, and hypertension [15, 17–21]. Furthermore, the NHI records of using expensive procedures and health services, as well as medications, are reliable, because the NHIA has an auditing system for preventing fraud [22]. The NHIA also has an inspection system for the NHI catastrophic illness registry system. As the NHI data generally have high quality, our research data on OHCA patients’ conditions regarding organ dysfunctions and diabetes are highly reliable.

### Statistical analysis

Because the secular patterns of OHCA incidence rates and mortality rates were both curvilinear, we estimated various linear and polynomial regression models for clarification of appropriate model specifications, as well as comparison purposes. As most curves of OHCA incidence rates and mortality rates reveal more than one point of local maximum, we estimated several general simple linear models and polynomial models with robust variance estimates that were based on White-corrected standard errors. We analyzed simple linear models, polynomial models adding the quadratic term of “year” and those adding both the quadratic term and the cubic term of “year.” We assessed the goodness of fit by referring to the statistical significance levels of independent variables and the levels of R-squared.

Social events, such as changes in policies, regulations and environments, may substantially influence the patterns of time-series data. To address the issue, we further conducted time-series data analysis. We tested several linear and polynomial regression specifications that model the annual rate (the number of incidences per 100,000 persons for the OHCA incidence rate, and the death hazard expressed in percentage for the OHCA mortality) in terms of a linear combination of the time period (year) as well as an autoregressive (AR) disturbance process for national data of Taiwan from 2000 to 2012. In each model, we analyzed the effects of the first-order, the second-order and the third-order autoregressive terms, which reflected impacts from past structural disturbances (the actual past values of dependent variable minus their expected past values according to the modeling). For assessing the goodness of fit, in addition to examining the statistical significance levels of independent variables, we referred to the size of the estimated standard deviation of the white-noise disturbance (sigma), which was the random part of variation in data. To address potential hetroskedastic problems, our modeling adopted robust variance estimators that are based on quasi-maximum likelihood estimation. This tended to expand the size of variance, and made our conclusions more conservative.

For OHCA patients with MV care, we examined the distribution of survival time, and compared demographic and clinical characteristics across patients with different lengths of survival time using the chi-squared test. We further used the logistic regression to identify factors associated with OHCA mortality among MV users. To address heterogeneity among hospitals, our logistic modeling adopted a robust variance estimator that adjusted for hospital-level intra-group correlation.

All our analyses adopted a significance level of 5%. For organizing data and constructing final analytical data files, we used SAS software version 9.1.3 (SAS Institute Inc., Cary, NC). Our model estimation was based on Stata software version 12 (StataCorp, College station, TX).

## Results

### The secular trend in OHCA incidence

**Fig 3:**
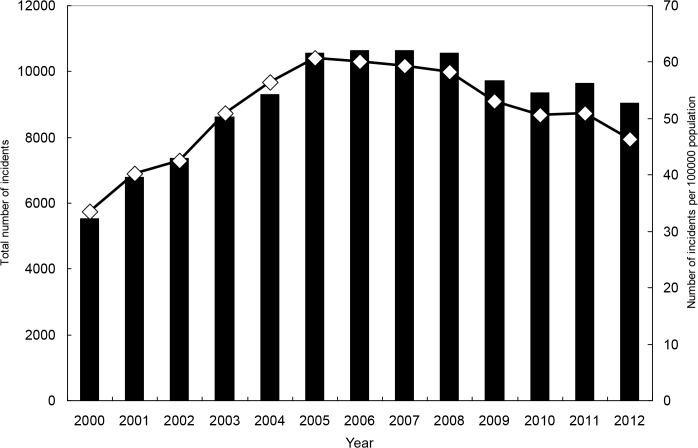
Total numbers of incidents with out-of-hospital cardiac arrest and incident rates, for both genders combined.

**Fig 4:**
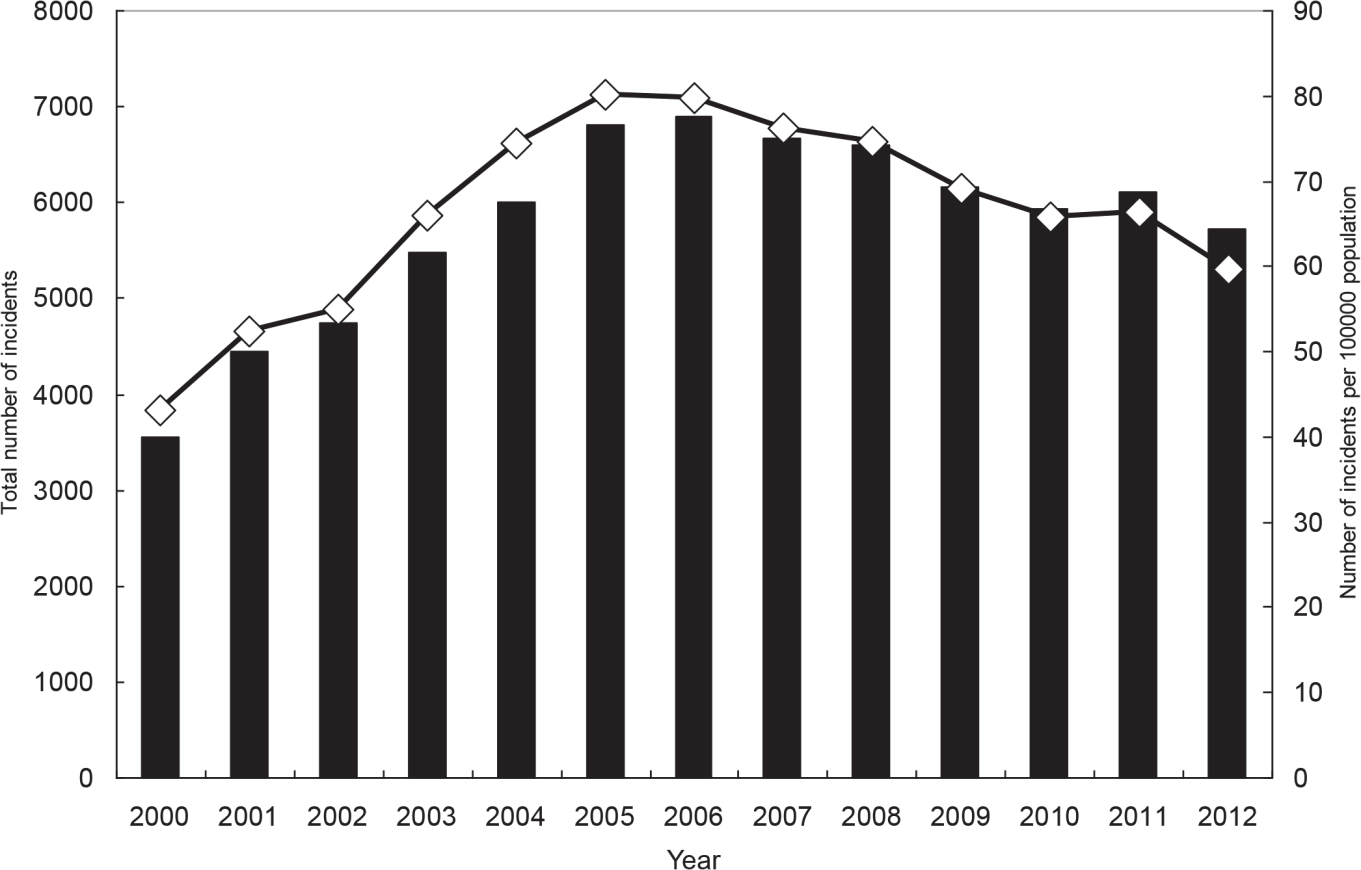
Total numbers of incidents with out-of-hospital cardiac arrest and incident rates, for men.

**Fig 5:**
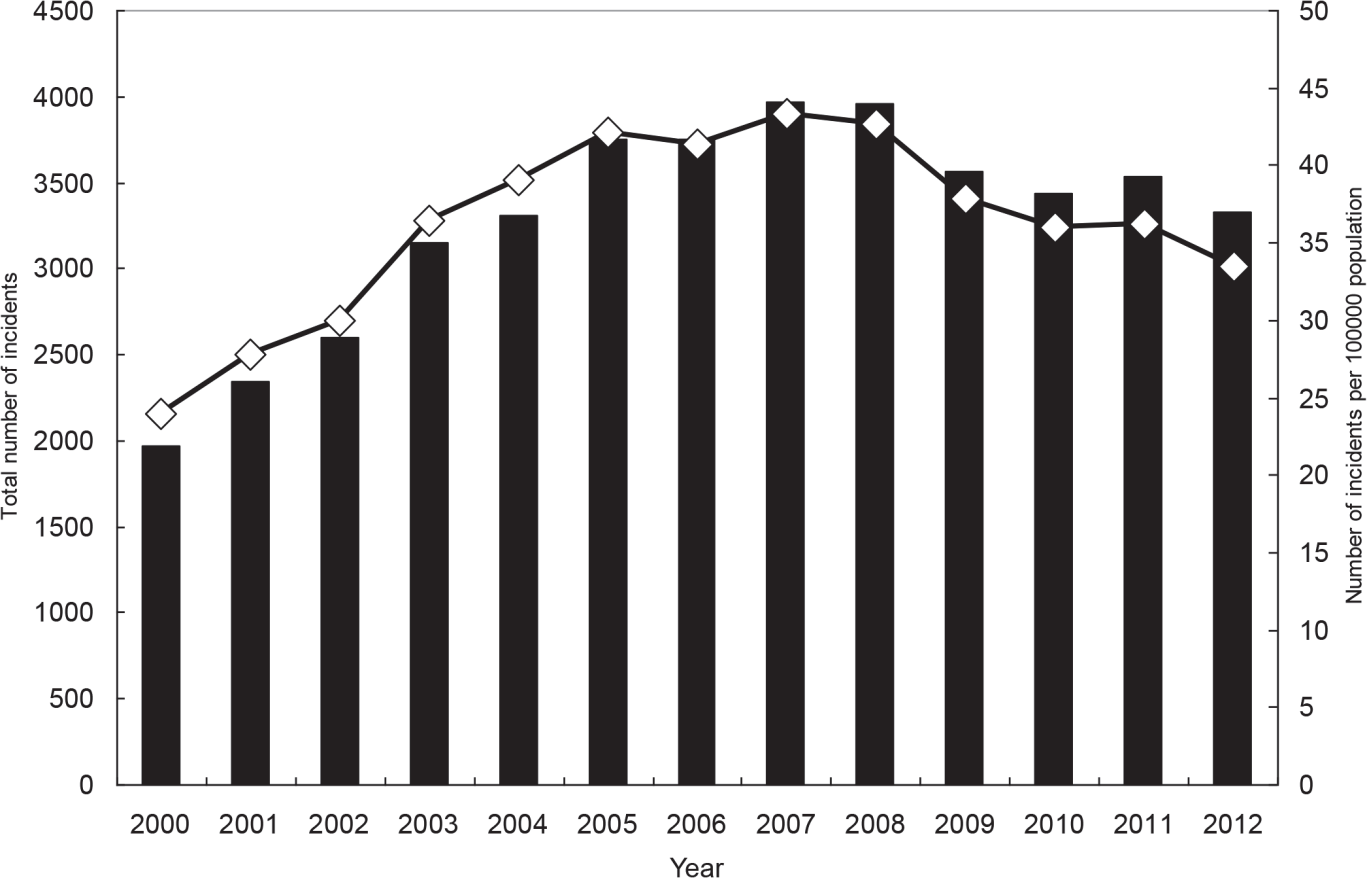
Total numbers of incidents with out-of-hospital cardiac arrest and incident rates, for women.

From 2000 to 2012, there were 117,787 OHCAs requiring CPR upon arrival at the hospital. The overall incidence rate during this period was 51.1 per 100,000 persons. For each gender and age range, the secular trend indicates a sharp increase in the early 2000s and a decrease afterwards (Tables 1 and 2, Figs 3–5, and S1–S4 Figs). For both genders combined, the incidence rate was 33.5 in year 2000, and it increased to 60.7 in 2005 and then declined to 46.3 in 2012. The rate for men was significantly higher than that for women, and the ratio was close to 2. Older age was strongly associated with higher hazard of OHCA.

**Table 1:** The secular trend in the OHCA incidence rate in Taiwan (the number of cases per 100,000 persons), for national data of Taiwan from 2000 to 2012, by gender.

	Both genders		Men		Women	
Year	IR	95% CI	IR	95% CI	IR	95% CI
2000	33.5	(32.7–34.4)	43.1	(41.7–44.5)	24.0	(22.9–25.0)
2001	40.2	(39.2–41.1)	52.3	(50.8–44.5)	27.8	(26.7–29.0)
2002	42.5	(41.6–43.5)	55.0	(53.5–56.6)	30.0	(28.9–31.2)
2003	50.9	(49.8–52.0)	66.0	(64.2–44.5)	36.5	(35.2–37.7)
2004	56.3	(55.2–57.5)	74.4	(72.5–76.3)	39.1	(37.8–40.4)
2005	60.7	(59.6–61.9)	80.2	(78.3–82.1)	42.2	(40.8–43.5)
2006	60.1	(59.061.3)	79.8	(78.0–81.7)	41.4	(40.0–42.7)
2007	59.4	(58.260.5)	76.3	(74.4–78.1)	43.3	(42.0–44.6)
2008	58.2	(57.1–59.3	74.6	(72.8–76.4)	42.6	(41.3–44.6)
2009	53.0	(52.0–54.1)	69.1	(67.4–70.8)	37.8	(36.6–39.1)
2010	50.5	(49.5–51.6)	65.9	(64.2–67.5)	36.0	(34.8–37.2)
2011	50.8	(49.8–51.9)	66.4	(64.7–68.0)	36.2	(35.0–37.4)
2012	46.3	(45.4–47.3)	59.6	(58.0–68.0)	33.5	(32.4–34.6)
Polynomial regression specifications^a^ that model the annual OHCA incidence rate (the number per 100,000 persons) in terms of a linear combination of the time period (t) as well as an autoregressive (AR) disturbance process, and adopt robust variance estimates *(refer to S3 and S4 Tables for more details)*						
	Coefficient	95% CI	Coefficient	95% CI	Coefficient	95% CI
Intercept	30.48^***^	(23.02–37.94)	39.25^***^	(28.44–50.06)	21.63^***^	(19.33–23.92)
t	9.94^***^	(5.22–14.66)	13.55^***^	(6.72–20.38)	6.52^***^	(4.84–8.21)
t^2^	-0.97^*^	(-1.76–0.18)	-1.38^*^	(-2.51–0.24)	-0.56^***^	(-0.87–0.25)
t^3^	0.02	(-0.02–0.06)	0.03	(-0.02–0.09)	0.01	(-0.01–0.02)
Lag 1	0.15	(-0.28–0.59)	0.19	(-0.24–0.62)	-0.21	(-0.75–0.33)
Lag 2	-0.51	(-1.71–0.69	-0.39	(-1.56–0.78)	-0.70^*^	(-1.25–1.43)
Lag 3	-0.40	(-1.120.31)	-0.54	(-1.32–0.23)	-0.38	(-0.81–0.06)
σ^b^	1.37^***^	(1.06–1.69)	1.74^***^	(0.29–2.18)	1.06^***^	(0.71–1.40)

^*^ p<0.05;

^**^p<0.01;

^***^p<0.001.

Abbreviations: CI, confidence interval; IR, incidence rate; OHCA, out-of-hospital cardiac arrest.

^a^For the year 2000, t = 0; t = 1 for the year 2001, t = 2 for the year 2002, and so on. The models include lags of 1, 2 and 3 of the structural disturbance.

^b^σ is *the estimated standard deviation of the white-noise disturbance*.

**Table 2:** The secular trend in the OHCA incidence rate in Taiwan (the number of cases per 100,000 persons), for national data of Taiwan from 2000 to 2012, by age.

Year	Aged 18~64		Aged 65~74		Aged 75~84		Aged 85+	
	IR	95%CI	IR	95%CI	IR	95%CI	IR	95%CI
2000	16.4	(15.7−17.0)	107.7	(101.9−113.4)	224	(211.9−236.1)	384.8	(350.2−419.4)
2001	19.3	(18.6−20.0)	129.6	(123.4−135.9)	258	(245.5−270.6)	503	(464.6−541.5)
2002	19.8	(19.1−20.5)	132.4	(126.1−138.7)	281	(268.4−293.7)	544.4	(505.9−582.9)
2003	22.8	(22.0−23.6)	150.7	(143.9−157.4)	328.3	(314.9−341.6)	670	(628.7−711.4)
2004	25.9	(25.1−26.7)	162.3	(155.3−169.3)	338	(324.8−351.3)	669.7	(629.1−710.2)
2005	28.1	(27.3−29.0)	169.9	(162.8−176.9)	364.3	(351.0−377.6)	711	(671.5−750.6)
2006	28.2	(27.3−29.0)	161	(154.2−167.8)	353.2	(340.3−366.1)	679.8	(643.0−716.6)
2007	25.9	(25.1−26.7)	155.5	(148.8−162.1)	371.9	(358.8−384.9)	666.7	(632.0−701.5)
2008	25	(24.3−25.8)	149.1	(142.6−155.5)	352.5	(339.9−365.0)	690.9	(656.8−724.9)
2009	22.6	(21.9−23.4)	137.4	(131.3−143.6)	314.5	(302.8−326.2)	602.6	(571.8−633.4)
2010	21.6	(20.9−22.3)	121.2	(115.4−127.0)	301.8	(290.4−313.2)	578.8	(550.0−607.7)
2011	22.1	(21.4−22.8)	127	(121.1−132.9)	294.5	(283.4−305.7)	540.1	(513.4−566.7)
2012	20.3	(19.6−21.0)	116.9	(111.3−122.5)	259.6	(249.2−270.0)	496.9	(472.2−521.6)
Polynomial regression specifications^a^ that model the annual OHCA incidence rate (the number per 100,000 persons) in terms of a linear combination of the time period (t) as well as an autoregressive (AR) disturbance process, and adopt robust variance estimates *(refer to S5 and S6 Tables for more details)*								
	Coefficient	95% CI	Coefficient	95% CI	Coefficient	95% CI	Coefficient	95% CI
Intercept	14.73^***^	(12.04−17.41)	98.86^***^	(87.08−110.63)	211.54^***^	(198.54−224.54)	375.34^***^	(359.76−390.92)
t	4.64^***^	(2.89−6.39)	29.4^***^	(22.08−36.72)	50.6^***^	(40.86−60.33)	132.28^***^	(122.60−141.96)
t^2^	-0.52^**^	(-0.83−0.22)	-3.99^***^	(-5.23−2.75)	-4.62^***^	(-6.47−2.77)	-16.05^***^	(-17.64−14.45)
t^3^	0.01	(-0.001−0.03)	0.14	(0.08−0.20)	0.05	(-0.04−0.15)	0.49^***^	(0.41−0.56)
Lag 1	0.28	(-0.18−0.73)	-0.19	(-0.65−0.27)	-0.47	(-1.02−0.09)	-1.18^***^	(-1.64−0.72)
Lag 2	-0.34	(-1.07−0.40)	-0.61	(-1.53−0.31)	-0.66^**^	(-1.17−0.15)	-0.97^***^	(-1.44−0.51)
Lag 3	-0.59^*^	(-1.17−0.01)	-0.45	(-1.07−0.17)	-0.54^**^	(-0.95−0.12)	-0.47^**^	(-0.83−0.12)
σ^b^	0.63^***^	(0.41−0.85)	4.01^***^	(2.61−5.42)	7.56^***^	(5.28−9.84)	10.72^***^	(5.87−15.58)

^*^ p<0.05;

^**^p<0.01;

^***^p<0.001.

Abbreviations: CI, confidence interval; IR, incidence rate; OHCA, out-of-hospital cardiac arrest.

^a^For the year 2000, t = 0; t = 1 for the year 2001, t = 2 for the year 2002, and so on. The models include lags of 1, 2 and 3 of the structural disturbance.

^b^σ is *the estimated standard deviation of the white-noise disturbance*.

Examining results from our general simple linear models and polynomial models with robust variance estimates (S3 and S4 Tables), we found that a polynomial model adding the quadratic term of “year” performed much better than its corresponding simple linear model, as the R-squared greatly increased after addition of the quadratic term. Adding the cubic term did not substantially increase the R-squared further. The results are consistent with the overall shape of a curve containing data points of annual incidence rates, which follows an inverted U-shaped function to some degree.

Results from corresponding time-series data analysis (S5 and S6 Tables) indicate significant second-order autoregressive effects for women and people aged 75 or older. Furthermore, results show that adding the cubic term effectively reduced the size of the white-noise disturbance (sigma), suggesting that addition of the cubic term had benefits in improving prediction power in models of time-series data analysis. Compared to the level of incidence rate, the size of remaining white-noise disturbance is modest.

### The secular trend in OHCA mortality

**Fig 6:**
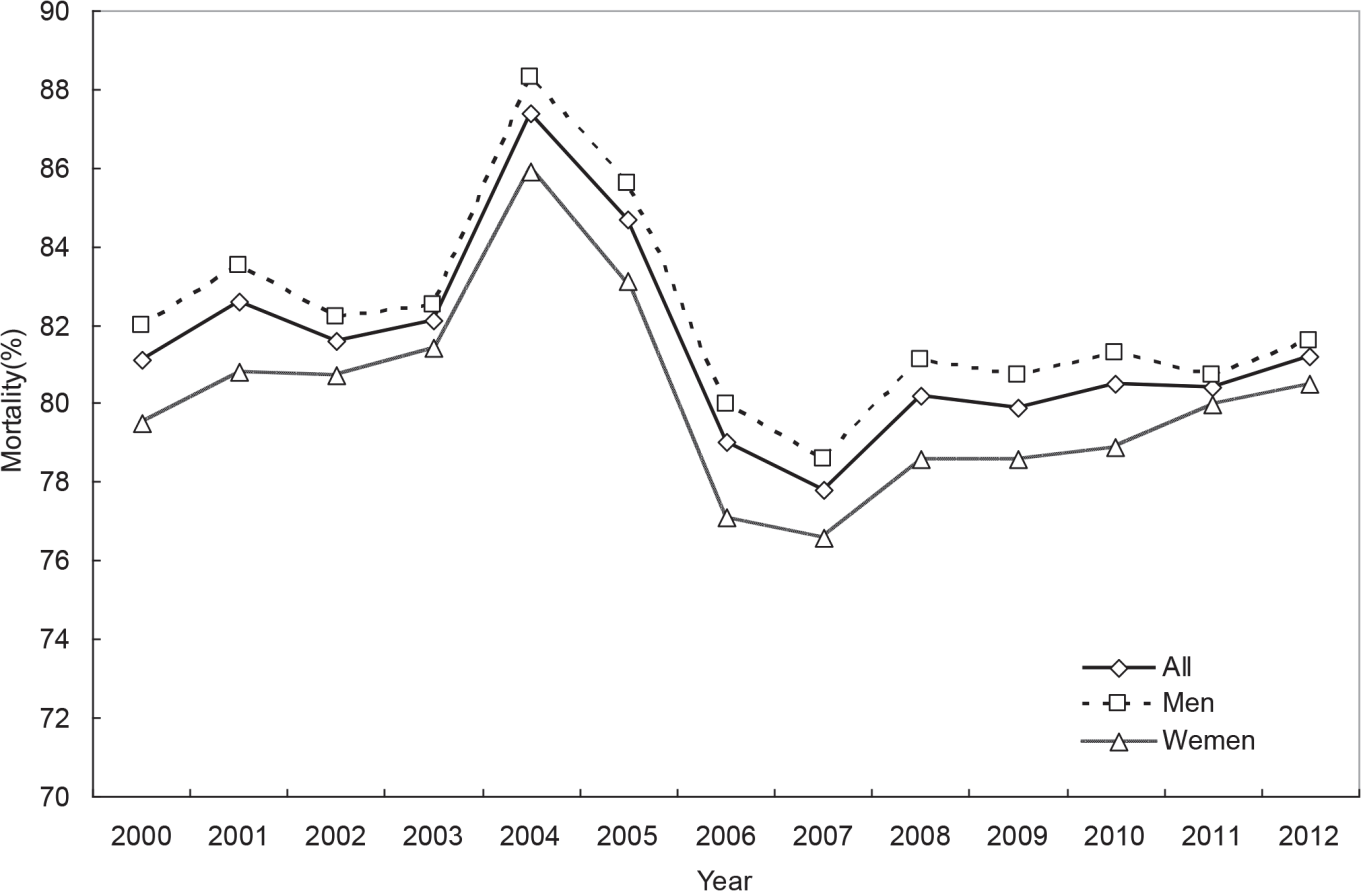
1-day mortality rates of out-of-hospital cardiac arrest, for both genders combined and by gender.

**Fig 7:**
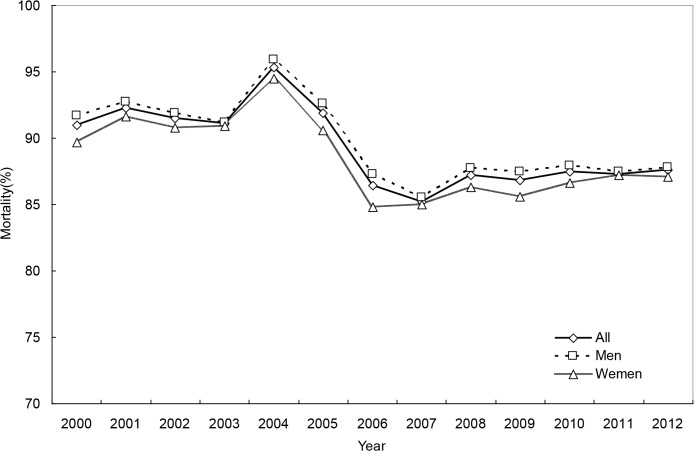
30-day mortality rates of out-of-hospital cardiac arrest, for both genders combined and by gender.

**Fig 8:**
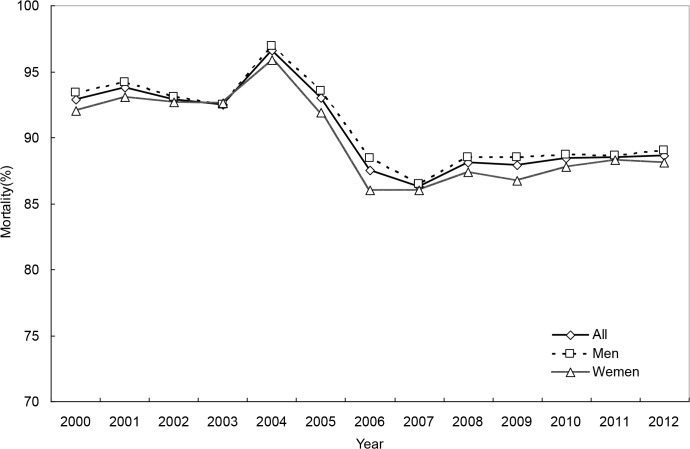
180-day mortality rates of out-of-hospital cardiac arrest, for both genders combined and by gender.

For the whole study period, 1-day, 30-day and 180-day OHCA mortality rates were 81.3%, 89.1%, and 90.2%, respectively. The trend in mortality was also curvilinear, revealing a substantial increase in the early 2000s, a subsequent steep decline and finally a modest increase (Tables 3 and 4, Figs 6–8, and S5–S7 Figs). Men and women had similar mortality levels. Mortality rates for various age groups were also comparable.

**Table 3:** The secular trend in the OHCA mortality rate (%), for national data of Taiwan from 2000 to 2012, by gender and survival time.

**Year**	**Both genders**						**Men**						**Women**					
****	**1-day MR**	**95% CI**	**30-day MR**	**95% CI**	**180-day MR**	**95% CI**	**1-day MR**	**95% CI**	**30-day MR**	**95% CI**	**180-day MR**	**95% CI**	**1-day MR**	**95% CI**	**30-day MR**	**95% CI**	**180-day MR**	**95% CI**
2000	**81.1**	(80.0–82.1)	**91**	(90.2–91.7)	**92.9**	(92.2–93.6)	**82**	(80.7–83.2)	**91.7**	(90.7–92.6)	**93.4**	(92.6–94.2)	**79.5**	(77.7–81.2)	**89.7**	(88.4–91.0)91.0)	**92**	(90.8–93.2)
2001	**82.6**	(81.7–83.5)	**92.3**	(91.7–92.9)	**93.8**	(93.2–94.4)	**83.5**	(82.4–84.6)	**92.7**	(91.9–93.4)	**94.2**	(93.5–94.8)	**80.8**	(79.2–82.4)	**91.6**	(90.5–92.7)	**93.1**	(92.1–94.2)
2002	**81.6**	(80.8–82.5)	**91.5**	(90.9–92.1)	**92.9**	(92.3–93.5)	**82.2**	(81.1–83.3)	**91.9**	(91.1–92.7)	**93.1**	(92.3–93.8)	**80.7**	(79.2–82.2)	**90.8**	(89.6–91.9)	**92.7**	(91.7–93.7)
2003	**82.1**	(81.3–82.9)	**91.1**	(90.5–91.7)	**92.5**	(91.9–93.5)	**82.5**	(81.5–83.5)	**91.2**	(90.5–92.0)	**92.5**	(91.8–93.2)	**81.4**	(80.0–82.7)	**90.9**	(89.9–91.9)	**92.6**	(91.7–93.5)
2004	**87.4**	(86.7–88.1)	**95.3**	(94.9–95.8)	**96.6**	(96.2–96.9)	**88.3**	(87.4–89.1)	**95.9**	(95.3–96.4)	**96.9**	(96.5–97.3)	**85.9**	(84.7–87.1)	**94.5**	(93.7–95.2)	**95.9**	(95.3–96.6)
2005	**84.7**	(84.0–85.4)	**91.9**	(91.4–92.4)	**93**	(92.5–93.4)	**85.6**	(84.8–86.4)	**92.6**	(92.0–93.2)	**93.5**	(93.0–94.1)	**83.1**	(81.9–84.3)	**90.6**	(89.7–91.6)	**91.9**	(91.0–92.8)
2006	**79**	(78.2–79.8)	**86.4**	(85.8–87.1)	**87.5**	(86.9–88.1)	**80**	(79.1–81.0)	**87.3**	(86.5–88.1)	**88.4**	(87.6–89.1)	**77.1**	(75.8–78.5)	**84.8**	(83.7–86.0)	**86**	(84.9–87.1)
2007	**77.8**	(77.0–78.6)	**85.2**	(84.6–85.9)	**86.3**	(85.6–86.9)	**78.6**	(77.6–81.0)	**85.5**	(84.6–86.3)	**86.5**	(85.6–87.3)	**76.6**	(75.2–77.9)	**85**	(83.8–86.0)	**86**	(84.9–87.1)
2008	**80.2**	(79.4–80.9)	**87.2**	(86.5–87.8)	**88.1**	(87.5–88.7)	**81.1**	(80.2–82.1)	**87.7**	(86.9–88.5)	**88.5**	(87.8–89.3)	**78.6**	(77.3–79.9)	**86.3**	(85.2–87.3)	**87.4**	(86.3–88.4)
2009	**79.9**	(79.1–80.7)	**86.8**	(86.1–87.5)	**87.9**	(87.2–88.5)	**80.7**	(79.7–81.6)	**87.5**	(86.7–88.3)	**88.5**	(87.7–89.3)	**78.6**	(77.3–79.9)	**85.6**	(84.5–86.8)	**86.7**	(85.6–87.8)
2010	**80.5**	(79.6–81.3)	**87.5**	(86.8–88.1)	**88.4**	(87.7–89.0)	**81.3**	(80.4–81.6)	**87.9**	(87.1–88.8)	**88.7**	(87.9–89.5)	**78.9**	(77.5–80.3)	**86.6**	(85.5–87.8)	**87.8**	(86.7–88.9)
2011	**80.4**	(79.7–81.2)	**87.3**	(86.7–88.0)	**88.5**	(87.8–89.1)	**80.7**	(79.7–81.7)	**87.5**	(86.7–88.3)	**88.6**	(87.8–89.4)	**80**	(78.6–81.3)	**87.2**	(86.0–88.2)	**88.3**	(87.2–89.3)
2012	**81.2**	(80.4–82.0)	**87.6**	(86.9–88.2)	**88.6**	(88.0–89.3)	**81.6**	(80.6–82.6)	**87.8**	(87.0–88.7)	**89**	(88.2–89.8)	**80.5**	(79.1–81.8)	**87.1**	(85.9–88.2)	**88.1**	(86.9–89.1)
Polynomial regression specificationsa that model the annual mortality rates (%) among OHCA patients in terms of a linear combination of the time period (t) as well as an autoregressive (AR) disturbance process, and adopt robust variance estimates (refer to S7 and S8 Tables for more details)																		
	**Both genders**						**Men**						**Women**					
	1day Coef.	95% CI	30day Coef.	95% CI	180day Coef.	95% CI	1day Coef.	95% CI	30day Coef.	95% CI	180day Coef.	95% CI	1day Coef.	95% CI	30day Coef.	95% CI	180day Coef.	95% CI
Int.	82.68*	(77.86-87.49)	90.09*	(88.35-91.83)	91.94*	(90.13-93.76)	79.80*	(77.84-81.75)	90.38*	(88.43-92.34)	92.03*	(90.14-93.92)	78.20*	(76.12-80.28)	89.30*	(87.59-91.00)	91.68*	(90.11-93.22)
t	3.21&	(0.39-6.02)	2.70#	(0.87-4.52)	2.46&	(0.51-4.41)	3.79*	(2.22-5.37)	2.80#	(1.04-4.56)	2.61#	(0.93-4.30)	3.60#	(1.38-5.82)	2.67#	(0.67-4.66)	2.29&	(0.21-4.36)
t^2^	-0.86*	(-1.33—0.39)	-0.74*	(-1.14—0.34)	-0.71#	(-1.14-0.29)	-0.83*	(-1.15-0.51)	-0.75*	(-1.12-0.38)	-0.74*	(-1.09-0.38)	-0.83#	(-1.30-0.35)	-0.74#	(-1.17-0.30)	-0.70#	(-1.16-0.23)
t^3^	0.05*	(0.03-0.07)	0.04*	(0.02-0.07)	0.04#	(0.02-0.07)	0.05*	(0.03-0.06)	0.04*	(0.02-0.06)	0.04*	(0.02-0.06)	0.05#	(0.02-0.07)	0.04#	(0.02-0.07)	0.04#	(0.01-0.07)
Lag1	-0.62	(-1.33-0.09)	-0.04	(-0.73-0.66)	-0.02	(-0.78-0.74)	-0.19	(-0.80-0.42)	-0.16	(-0.87-0.54)	-0.16	(-0.87-0.55)	0.05	(-0.84-0.94)	0.13	(-0.38-0.63)	0.13	(-0.44-0.71)
Lag2	-0.47	(-1.31-0.37)	-0.65*	(-0.93—0.36)	-0.64*	(-0.90-0.38)	-0.73*	(-0.94-0.51)	-0.64*	(-0.88-0.39)	-0.64*	(-0.87-0.42)	-0.69*	(-0.92-0.45)	-0.66#	(-1.03-0.28)	-0.63#	(-0.99-0.26)
Lag3	-0.19	(-0.89-0.51)	-0.31	(-0.87-0.26)	-0.31	(-0.91-0.28)	-0.42	(-0.93-0.08)	-0.45	(-0.99-0.09)	-0.46	(-1.00-0.07)	-0.24	(-1.00-0.52)	-0.06	(-0.50-0.38)	-0.13	(-0.65-0.39)
σ^b^	2.19*	(1.81-2.56)	1.09*	(0.69-1.49)	1.09*	(0.69-1.49)	1.03*	(0.72-1.34)	1.06*	(0.71-1.42)	0.99*	(0.65-1.34)	1.07*	(0.74-1.40)	1.19*	(0.71-1.67)	1.21*	(0.71-1.70)

^&^p<0.05;

^#^p<0.01;

^*^p<0.001.

Abbreviations: CI, confidence interval; Coef., coefficient; Int., intercept; MR, mortality rate; OHCA, out-of-hospital cardiac arrest.

^a^For the year 2000, t = 0; t = 1 for the year 2001, t = 2 for the year 2002, and so on. The models include lags of 1, 2 and 3 of the structural disturbance.

^b^σ is *the estimated standard deviation of the white-noise disturbance*

**Table 4:** The secular trend in the OHCA mortality rate in Taiwan from 2000 to 2012 (%), by age and survival time.

Year	**Aged 18-64**						**Aged 65-74**						**Aged 75-84**						**Aged 85+**					
	**1-day MR**	95% CI	**30-day MR**	95% CI	**180-day MR**	95% CI	**1-day MR**	95% CI	**30-day MR**	95% CI	**180-day MR**	95% CI	**1-day MR**	95% CI	**30-day MR**	95% CI	**180-day MR**	95% CI	**1-day MR**	95% CI	**30-day MR**	95% CI	**180-day MR**	95% CI
2000	**78.5**	(76.9-80.2)	**90.7**	(89.6-91.9)	**92.3**	(91.3-93.4)	**84.2**	(82.3-86.1)	**93.2**	(91.9-94.5)	**95.4**	(94.3-96.5)	**83.8**	(81.8-85.8)	**91.3**	(89.8-92.8)	**93.6**	(92.2-94.9)	**77.2**	(73.4-80.9)	**84.8**	(81.5-88.0)	**86.7**	(83.6-89.7)
2001	**80.2**	(78.8-81.7)	**92.3**	(91.3-93.3)	**93.8**	(93.0-94.7)	**84.1**	(82.4-85.9)	**92.9**	(91.7-94.2)	**94.5**	(93.4-95.6)	**86.7**	(85.0-88.3)	**94.1**	(92.9-95.2)	**95.5**	(94.4-96.5)	**78.8**	(75.6-81.9)	**86.4**	(83.8-89.0)	**87.8**	(85.3-90.3)
2002	**79.1**	(77.7-80.6)	**90.3**	(89.3-91.4)	**91.8**	(90.8-92.8)	**83**	(81.2-84.7)	**93**	(91.8-94.2)	**94.1**	(93.0-95.3)	**84.7**	(83.1-86.3)	**92.9**	(91.8-94.1)	**94.7**	(93.7-95.7)	**81.2**	(78.4-83.9)	**89.1**	(86.9-91.3)	**90.3**	(88.2-92.4)
2003	**79.1**	(77.8-80.5)	**89.1**	(88.1-90.2)	**90.3**	(89.3-91.3)	**83.4**	(81.7-85.1)	**92.8**	(91.7-94.0)	**94.5**	(93.5-95.6)	**85.1**	(83.6-86.5)	**92.8**	(91.8-93.9)	**94.2**	(93.3-95.2)	**82.7**	(80.4-85.1)	**90.6**	(88.8-92.4)	**91.9**	(90.2-93.6)
2004	**86.2**	(85.1-87.3)	**94.8**	(94.1-95.5)	**95.7**	(95.1-96.4)	**88.3**	(86.9-89.7)	**96.2**	(95.4-97.0)	**97.3**	(96.6-98.0)	**88.5**	(87.2-89.7)	**95.7**	(94.9-96.5)	**97.4**	(96.8-98.0)	**87.6**	(85.6-89.6)	**94.8**	(93.5-96.2)	**96.1**	(94.9-97.2)
2005	**82.6**	(81.4-83.7)	**91.1**	(90.2-92.0)	**92**	(91.2-92.8)	**86.1**	(84.6-87.5)	**92.6**	(91.5-93.7)	**93.6**	(92.5-94.6)	**86.3**	(85.1-87.6)	**92.8**	(91.8-93.7)	**94**	(93.2-94.9)	**86**	(84.0-87.9)	**91.5**	(89.9-93.0)	**92.8**	(91.3-94.2)
2006	**77.4**	(76.2-78.7)	**86.5**	(85.5-87.5)	**87.5**	(86.5-88.4)	**78.4**	(76.6-80.1)	**85.9**	(84.4-87.4)	**87.3**	(85.9-88.7)	**81**	(79.6-82.4)	**86.5**	(85.2-87.7)	**87.5**	(86.2-88.7)	**80.8**	(78.6-82.9)	**87**	(85.2-88.8)	**88.3**	(86.6-90.1)
2007	**75.7**	(74.3-77.0)	**83.9**	(82.8-85.0)	**84.7**	(83.6-85.8)	**76.2**	(74.4-78.0)	**84.9**	(83.3-86.4)	**86.1**	(84.6-87.6)	**80.5**	(79.1-81.9)	**86.8**	(85.6-87.9)	**87.9**	(86.8-89.1)	**80.5**	(78.4-82.6)	**86.3**	(84.5-88.1)	**87.3**	(85.6-89.1)
2008	**78.9**	(77.6-80.2)	**86.7**	(85.6-87.7)	**87.4**	(86.4-88.4)	**78.8**	(77.0-80.6)	**86.5**	(85.1-88.0)	**87.8**	(86.4-89.2)	**81.3**	(79.9-82.7)	**87.2**	(86.0-88.4)	**88.2**	(87.1-89.4)	**83.1**	(81.2-84.9)	**89**	(87.4-90.5)	**90**	(88.5-91.5)
2009	**78.4**	(77.0-79.7)	**86**	(84.8-87.1)	**87**	(85.9-88.1)	**78.3**	(76.4-80.1)	**86.3**	(84.7-87.8)	**87.4**	(85.9-88.9)	**82.4**	(80.9-83.8)	**88.1**	(86.9-89.3)	**89.1**	(87.9-90.3)	**81.2**	(79.2-83.2)	**87.2**	(85.5-88.9)	**88.4**	(86.8-90.0)
2010	**79.9**	(78.6-81.3)	**87.3**	(86.2-88.4)	**88**	(86.9-89.1)	**78.3**	(76.3-80.3)	**86.6**	(85.0-88.2)	**87.7**	(86.1-89.2)	**81.9**	(80.4-83.3)	**88.1**	(86.9-89.3)	**89.1**	(87.9-90.2)	**81.5**	(79.5-83.4)	**87.8**	(86.1-89.4)	**88.9**	(87.3-90.4)
2011	**79**	(77.7-80.3)	**86.7**	(85.6-87.8)	**87.7**	(86.6-88.8)	**79.3**	(77.4-81.2)	**86.5**	(84.9-88.1)	**87.8**	(86.3-89.3)	**81.5**	(80.1-83.0)	**87.9**	(86.7-89.2)	**89.1**	(87.9-90.3)	**83.3**	(81.4-85.1)	**88.8**	(87.3-90.4)	**89.7**	(88.2-91.2)
2012	**79.5**	(78.2-80.9)	**86.2**	(85.1-87.4)	**87.5**	(86.4-88.6)	**80.3**	(78.4-82.2)	**88.1**	(86.6-89.7)	**89**	(87.5-90.5)	**82.2**	(80.7-83.7)	**88.1**	(86.8-89.4)	**89.3**	(88.0-90.5)	**84.4**	(82.6-86.2)	**89.1**	(87.5-90.6)	**89.9**	(88.4-91.4)
Polynomial regression specifications^a^ that model the annual mortality rates (%) among OHCA patients in terms of a linear combination of the time period (t) as well as an autoregressive (AR) disturbance process, and adopt robust variance estimates *(refer to S9 and S10 Tables for more details)*																								
	**Aged 18-64**						**Aged 65-74**						**Aged 75-84**						**Aged 85+**					
	1day Coef.	95% CI	30day Coef.	95% CI	180day Coef.	95% CI	1day Coef.	95% CI	30day Coef.	95% CI	180day Coef.	95% CI	1day Coef.	95% CI	30day Coef.	95% CI	180day Coef.	95% CI	1day Coef.	95% CI	30day Coef.	95% CI	180day Coef.	95% CI
Int.	76.32^*^	(73.78−78.86)	89.31^*^	(87.00−91.63)	90.98^*^	(88.72−93.23)	81.43^*^	(78.74−84.11)	91.91^*^	(90.11−93.71)	94.13^*^	(92.55−95.72)	82.99^*^	(81.15−84.82)	91.24^*^	(89.68−92.80)	93.44^*^	(92.05−94.82)	74.99^*^	(72.45−77.54)	83.80^*^	(81.98−85.62)	85.51^*^	(83.56−87.46)
t	3.99^*^	(1.99−5.99)	2.55^＃^	(0.70−4.39)	2.39^＃^	(0.63−4.15)	3.85^＃^	(1.57−6.14)	2.80^＃^	(1.02−4.58)	2.25^＃^	(0.59−3.90)	2.97^*^	(1.62−4.32)	2.70^＃^	(1.16−4.24)	2.42^＃^	(0.74−4.10)	5.67^*^	(3.74−7.60)	4.91^*^	(3.07−6.74)	4.71^*^	(2.84−6.57)
t^2^	-0.85^*^	(-1.25−0.44)	-0.70^*^	(-1.07−0.32)	-0.70^*^	(-1.06−0.34)	-0.99^*^	(-1.45−0.53)	-0.86^*^	(-1.23−0.48)	-0.76^*^	(-1.12−0.41)	-0.73^*^	(-0.99−0.46)	-0.75^*^	(-1.07−0.42)	-0.73^*^	(-1.11−0.36)	-1.03^*^	(-1.40−0.65)	-0.95^*^	(-1.35−0.56)	-0.93^*^	(-1.32−0.53)
t^3^	0.05^*^	(0.02−0.07)	0.04^*^	(0.02−0.06)	0.04^*^	(0.02−0.06)	0.06^*^	(0.03−0.08)	0.05^*^	(0.03−0.07)	0.05^*^	(0.03−0.07)	0.04^*^	(0.03−0.06)	0.04^*^	(0.02−0.06)	0.04^*^	(0.02−0.06)	0.05^*^	(0.03−0.07)	0.05^*^	(0.03−0.07)	0.05^*^	(0.03−0.07)
Lag1	-0.29	(-0.87−0.28)	-0.32	(-0.93−0.30)	-0.33	(-0.95−0.29)	-0.02	(-0.89−0.86)	-0.02	(-0.71−0.68)	0.09	(-0.57−0.75)	0.05	(-0.24−0.35)	0.09	(-0.27−0.45)	0.04	(-0.46−0.54)	-0.31	(-1.18−0.56)	-0.16	(-0.83−0.51)	-0.16	(-0.82−0.50)
Lag2	-0.67^*^	(-0.95−0.38)	-0.65^*^	(-0.98−0.31)	-0.64^*^	(-0.96−0.33)	-0.73^*^	(-0.91−0.55)	-0.63^*^	(-0.89−0.38)	-0.67^*^	(-0.91−0.43)	-0.76^*^	(-1.14−0.38)	-0.63^＃^	(-1.08−0.17)	-0.60^＃^	(-1.05−0.15)	-0.69^*^	(-0.92−0.46)	-0.55^*^	(-0.84−0.25)	-0.54^*^	(-0.81−0.27)
Lag3	-0.51^＃^	(-0.94−0.09)	-0.51^＆^	(-0.95−0.06)	-0.53^＆^	(-0.96−0.09)	-0.33	(-1.10−0.44)	-0.4	(-1.04−0.24)	-0.35	(-0.99−0.29)	-0.18	(-0.68−0.32)	-0.18	(-0.53−0.17)	-0.23	(-0.62−0.15)	-0.41	(-1.21−0.38)	-0.44	(-1.08−0.20)	-0.49	(-1.10−0.12)
σ^b^	1.30^*^	(0.90−1.69)	1.26^*^	(0.87−1.65)	1.20^*^	(0.81−1.59)	1.10^*^	(0.75−1.46)	1.01^*^	(0.74−1.28)	0.90^*^	(0.65−1.15)	0.89^*^	(0.63−1.15)	1.16^*^	(0.79−1.53)	1.20^*^	(0.77−1.64)	1.03^*^	(0.72−1.33)	1.12^*^	(0.79−1.44)	1.08^*^	(0.75−1.41)

^&^p<0.05;

^#^p<0.01;

*p<0.001.

Abbreviations: CI, confidence interval; Coef., coefficient; Int., intercept; MR, mortality rate; OHCA, out-of-hospital cardiac arrest.

^a^For the year 2000, t = 0; t = 1 for the year 2001, t = 2 for the year 2002, and so on. The models include lags of 1, 2 and 3 of the structural disturbance.

^b^σ is *the estimated standard deviation of the white-noise disturbance*.

Results from general “simple” linear models of mortality suggest that both the 30-day and 180-day mortality rates had a long-term decreasing trend over the period (all *p-values*<0.01; S7 and S8 Tables). In contrast, the results indicate that 1-day mortality did not have a long-term declining trend over the 13 years. Unlike results from analyzing incidence rates, adding the quadratic term into the simple linear model did not substantially improve the value of R-squared, but further adding the cubic term greatly increased the R-squared. This suggests that the final model was more appropriate. Generally speaking, the explanation power of modeling the mortality level is not as good as that of modeling incidence rate.

Results from corresponding time-series data analysis (S9 and S10 Tables) reveal that significant second-order autoregressive effects also emerged for mortality. Results also tend to suggest that adding the cubic term may decrease the size of the white-noise disturbance. Compared to the mortality level, the size of remaining white-noise disturbance is modest.

### Epidemiologic features and outcomes among OHCA patients using MV

Among the 2010–2011 cohort of OHCA patients requiring CPR and using MV care in the hospital, 1-day, 30-day and 180-day OHCA mortality rates were 31.3%, 75.8%, and 86.0%, respectively (Table 5). The 1-day mortality rate for this OHCA cohort using both CPR and MV care in the hospital was much lower than that for OHCA patients with CPR but without MV care in the hospital. However, the differences regarding 30-day and 180-day mortality rates were not as salient, particularly the 180-day mortality rate.

**Table 5:** Demographic and clinical characteristics among OHCA patients requiring MV care during the hospital stay in 2010–2011, by the length of survival time.

			The length of survival time						
	All		< = 1 day		2–30 days		> 30 days		P-value
	N = 2105		N = 658		N = 937		N = 510		
	n	%	n	%	n	%	n	%	
*1-day mortality*	658	31.26							
*30-day mortality*	1595	75.77							
*180-day mortality*	1810	85.99							
***Age (in years)***									0.344
18–64	761	36.15	223	33.89	337	35.97	201	39.41	
65–74	445	21.14	133	20.21	206	21.99	106	20.78	
75–84	582	27.65	196	29.79	249	26.57	137	26.86	
85+	317	15.06	106	16.11	145	15.47	66	12.94	
***Gender***									*0*.*033*
Male	1253	59.52	388	58.97	537	57.31	328	64.31	
Female	852	40.48	270	41.03	400	42.69	182	35.69	
***Charlson comorbidity index***									0.061
0	931	44.23	280	42.55	410	43.76	241	47.25	
1	471	22.38	150	22.8	204	21.77	117	22.94	
2	276	13.11	84	12.77	117	12.49	75	14.71	
> = 3	427	20.29	144	21.88	206	21.99	77	15.10	
***Major surgery along with MV use***									
Any surgery	118	5.61	30	4.56	59	6.30	29	5.69	0.331
Cardiopulmonary	94	4.47	23	3.50	46	4.91	25	4.90	0.348
Gastrointestinal	2	0.10	1	0.15	1	0.11	0	0.00	0.697
Hepatobiliary & Pancreas	2	0.10	2	0.30	0	0.00	0	0.00	0.111
Urogenital	4	0.19	3	0.46	1	0.11	0	0.00	0.152
Neurology	19	0.90	4	0.61	11	1.17	4	0.78	0.475
Others	19	0.90	8	1.22	6	0.64	5	0.98	0.478
***Organ dysfunction Prior to the index admission***									
Heart failure	81	3.85	27	4.10	36	3.84	18	3.53	0.880
Renal failure	144	6.84	41	6.23	72	7.68	31	6.08	*0*.*388*
Hepatic failure	20	0.95	15	2.28	4	0.43	1	0.20	*0*.*0001*
Stroke	116	5.51	47	7.14	42	4.48	27	5.29	0.070
Severe obstructive airway diseases	219	10.40	65	9.88	104	11.10	50	9.80	0.645
***Diabetes mellitus prior to the index admission***									
Insulin	162	7.70	46	6.99	71	7.58	45	8.82	0.499
Oral hypoglycemic agent	338	16.06	85	12.92	175	18.68	78	15.29	*0*.*007*
***Cancer prior to the index admission***									
Any cancer	184	8.74	74	11.25	77	8.22	33	6.47	*0*.*012*
Lung	27	1.28	10	1.52	16	1.71	1	0.20	*0*.*041*
Breast	11	0.52	3	0.46	4	0.43	4	0.78	0.640
Colon and rectum	25	1.19	10	1.52	11	1.17	4	0.78	0.515
Esophagus	7	0.33	3	0.46	1	0.11	3	0.59	0.253
Stomach	9	0.43	5	0.76	2	0.21	2	0.39	0.255
Hepatobiliary	16	0.76	7	1.06	8	0.85	1	0.20	0.216
Leukemia	1	0.05	0	0.00	1	0.11	0	0.00	0.536
Lymphoma	6	0.29	4	0.61	1	0.11	1	0.20	0.165
Ovary, uteri and cervix	6	0.29	3	0.46	2	0.21	1	0.20	0.611
Oral cavity and neck	29	1.38	9	1.37	11	1.17	9	1.76	0.654
Other cancer	55	2.61	24	3.65	21	2.24	10	1.96	0.127
***Hospital type***									*<*.*0001*
Medical center	537	25.51	112	17.02	246	26.25	179	35.10	
Regional hospital	1226	58.24	427	64.89	563	60.09	236	46.27	
Local hospital	342	16.25	119	18.09	128	13.66	95	18.63	
***Location of the patient’s NHI registration***									*<*.*0001*
Big city	983	46.70	264	40.12	448	47.81	271	53.14	
Small city or town	736	34.96	241	36.63	340	36.29	155	30.39	
Remote or rural area	386	18.34	153	23.25	149	15.9	84	16.47	

Abbreviations: MV, mechanical ventilation; NHI, national health insurance; OHCA, out-of-hospital cardiac arrest.

The chi-squared tests indicate that several characteristics were negatively associated with the length of survival time (Table 5). They included the female gender, liver failure and cancer (all *p*-values < 0.05). Patients delivered to regional hospitals appeared to have a shorter length of survival time (*p* < 0.001). Patients residing in non-metropolitan areas also had worse survival chance (*p* < 0.001).

Our multiple logistic regression models yielded similar results, as indicated by adjusted odds ratios (ORs) in Table 6. Women tended to have worse 1-month and 6-month survival rates in this OHCA cohort with MV use (both *p-values* < 0.05). Cancer was a strong risk for 1-day survival (adjusted OR = 2.23; *p* < 0.01). An age greater ≥ 75 and a Charlson comorbidity index score ≥ 2 appeared to be significant risk factors for 6-month survival (all *p-values* < 0.05). Outcomes were better in metropolitan areas than in less urban areas (most corresponding *p-values* < 0.05). Compared to OHCA patients delivered to medical centers, those delivered to regional hospitals or local hospitals appeared to have a higher 1-day mortality (both *p-values* < 0.01), and OHCA patients receiving CPR and MV care in regional hospitals also tended to have higher 1-month and 6-month mortality rates (both *p-values* < 0.001).

**Table 6:** Factors associated with 1-day, 1-month and 6-month mortality rates among OHCA patients requiring MV care during the hospital stay: logistic regression models that adopt a robust variance estimator adjusting for hospital-level intra-group correlation.

	1-day mortality		1-month mortality		6-month mortality	
Explanatory variable	Adjusted OR	95% CI	Adjusted OR	95% CI	Adjusted OR	95% CI
**Age (ref: 18-64years old)**						
65–74	1.3	(0.74-2.30)	1.09	(0.78-1.52)	1.25	(0.85-1.84)
75–84	1.5	(0.98-2.29)	1.09	(0.80-1.48)	***1.41*** *	(1.00-2.00)
85+	1.39	(0.79-2.44)	1.28	(0.91-1.79)	***2.02*** **	(1.29-3.14)
**Gender (ref: female)**						
Male	1.1	(0.77-1.57)	***0.76*** **	(*0.62-0.93*)	***0.76*** *	(0.58-0.99)
**Charlson comorbidity index (ref: 0–1)**						
Charlson comorbidity index > = 2	0.97	(0.68-1.38)	1.12	(0.88-1.43)	***1.51*** *	(1.10-2.08)
**Major surgery along with MV use (ref: no such surgery)**						
Cardiopulmonary	0.94	(0.41-2.14)	0.96	(0.56-1.67)	0.73	(0.45-1.16)
Other than cardiopulmonary	1.48	(0.27-8.16)	2.24	(0.81-6.22)	1.93	(0.56-6.66)
**Heart failure**						
Prior to the index admission	0.13	(0.62-1.01)	0.97	(0.56-1.66)	1.34	(0.58-3.09)
**Renal failure**						
Prior to the index admission	1.2	(0.60-2.39)	1.18	(0.76-1.84)	1.6	(0.84-3.04)
**Hepatic failure**						
Prior to the index admission	2.32	(0.71-7.62)	6.57	(0.89-48.41)	2.91	(0.36-23.22)
**Stroke**						
Prior to the index admission	1.43	(0.79-2.59)	1.04	(0.63-1.71)	0.88	(0.46-1.69)
**Severe obstructive airway diseases**						
Prior to the index admission	0.67	(0.40-1.11)	0.99	(0.69-1.41)	1.29	(0.77-2.18)
**Diabetes mellitus**						
Insulin	0.88	(0.42-1.86)	0.74	(0.52-1.06)	0.98	(0.57-1.71)
Oral hypoglycemic agent	*0.50* **	(*031-0.81*)	1.03	(0.77-1.38)	1.18	(0.76-1.82)
**Cancer (ref: no cancer)**						
Any cancer	***2.23*** **	(*1.34-3.68*)	1.35	(0.91-2.01)	1.78	(0.98-3.25)
**Hospital type (ref: medical center)**						
Regional hospital	***3.83*** **	(*1.40-10.45*)	***2.05*** ***	(1.54-2.73)	***1.95*** ***	(1.49-2.54)
Local hospital	***5.15*** **	(*1.81-14.66*)	1.27	(0.91-1.78)	1.05	(0.72-1.52)
**NHI registration location (ref: big city)**						
Small city or town	1.03	(0.70-1.63)	***1.36*** **	(*1.13-1.64*)	***1.51*** **	(1.17-1.95)
Remote or rural area	***1.82*** *	(*1.11-2.98*)	1.26	(0.94-1.68)	***1.51*** *	(1.05-2.18)

^*^ p<0.05;

^**^ p<0.01;

^***^ p<0.001.

Abbreviations: CI, confidence interval; MV, mechanical ventilation; OHCA, out-of-hospital cardiac arrest; OR, odds ratio.

## Discussion

This nationwide population-based study in Taiwan yielded several significant findings. First, the overall incidence rate of OHCA requiring CPR upon arrival at the hospital in Taiwan during the period from 2000 to 2012 was 51.1 per population of 100,000 persons, and the rate was 46.3 in 2012. The secular trend in the incidence rate varied in different study periods, which was generally upward between 2000 and 2005, but downward between 2006 and 2012.

A recent study in Australia reported an OHCA incidence rate reflecting 52.6 events per 100,000 person-years in 2004–2005, and a decreasing trend leading to a rate of 48.4 events per 100,000 person-years in 2009–2010 [23]. In contrast, a recent study in South Korea showed that the OHCA incidence rate increased from 37.5 events per 100,000 person-years in 2006 to 46.8 in 2010 [24]. While the OHCA incidence rates in these countries had substantial differences in the mid-2000s, they seemed to be comparable in recent years, suggesting a trend toward more similar patterns of OHCA prevention and hospital care delivery across countries.

Regarding OHCA incidence rates across different gender and age groups, we found that the rate for men were about twice that for women, and persons aged 75 or older had a much higher risk from OHCA than younger adults. These findings suggest that men and elderly people 75 years of age or older may be particular focus groups for OHCA prevention.

Second, our study found that the overall 1-day, 30-day and 180-day mortality rates of all OHCA patients receiving CPR upon arrival at the hospital in 2000–2012 were respectively 81.3%, 89.1%, and 90.2%, and the 30-day and 180-day mortality rates had a long-term decreasing trend over the period while both their trend patterns were curvilinear (a substantial increase in the early 2000s, a subsequent steep decline and finally a modest increase).

One U.S. study in 2011 showed the survival rate to hospital admission of OHCA patients with cardiac causes and pre-hospital CPR was 26.3%, and the overall survival rate to hospital discharge was 9.6% [25]. A study reviewing global OHCA survival rates for years spanning from the early 1990s to the mid-2000s reported that the rates of survival to discharge among OHCA patients delivered to emergency care facilities were 6%, 9%, and 11% in North America, Europe, and Australia, respectively [26]. Regarding the time trend in OHCA survival chance, a study in Sweden indicated that the 1-month survival rate of OHCA witnessed in the emergency care center significantly increased from 13.9% in 1992 to 21.8% in 2009 [27]. In contrast, a U.S. study showed that the survival rate to hospital discharge among OHCA patients receiving no CPR before arrival at the hospital or getting out-of-hospital CPR from laypersons also significantly increased over time, from 3.7% in 2005 to 9.8% in 2009 [28].

These findings in the literature suggest that the chance of survival to hospital discharge or one month among OHCA patients has substantially improved over time globally, regardless of various settings of pre-hospital care or definitions of OHCA. This might reflect improvement of post-resuscitation care over recent years, particularly in hospitals with more resource and training. Our findings also show that the difference in 1-day mortality across OHCA patients with different recruitment criteria was more apparent than those with respect to mid-term or long-term mortality rates, and the 1-day mortality rate for all OHCA patients requiring CPR upon arrival at the hospital had no apparent improvement from the early 2000s to the early 2010s. This might imply that more effort should be devoted into advances in the process of resuscitation. This also tends to imply that further advancing the process of resuscitation might be a more difficult challenge than improving post-resuscitation care.

Third, our study found that 31.3% of OHCA patients requiring both CPR and MV care in the hospital died within 24 hour after hospital admission, and only 24.2% and 14.0% could survive at least one month and six months, respectively. Previous studies showed that outcomes after cardiac arrest can be affected by multi-factors, including the time of return of spontaneous circulation (ROSC), initial cardiac rhythm, age and comorbidities [29–31]. Our data for the OHCA cohort using both CPR and MV care did show that older age and certain morbid conditions (as reflected by the Charlson comorbidity index) were significantly associated with a lower chance of survival to six months. The findings suggest that poor outcomes were very likely to eventually develop in these subgroups of survivors after having cardiac arrest and subsequently receiving intensive care for a relatively long time. Because decisions upon treatment for OHCA patients should take into account patients’ own wishes and welfare, more effort into improving knowledge on post-arrest prognosis and advocating advanced care planning is warranted [32, 33].

Fourth, we noted that OHCA patients delivered to medical centers and those residing in metropolitan areas tended to have better survival chance. The findings are reasonable, as metropolitan areas have more affluent healthcare resources for both pre-hospital services and post-resuscitation care, and medical centers may have more advanced equipments and training courses that are effective in improving post-resuscitation care. It remains a great challenge to narrow such disparities. Innovative ways are necessary to reduce such disparities caused by the inherent unequal distribution in resource across healthcare organizations and residential areas.

Our study also reveals a question in regard to gender difference in health outcomes. Although the unadjusted 30-day and 180-day mortality rates of female OHCA patients requiring CPR upon arrival at the hospital were comparable to those of their male counterparts, female OHCA patients requiring both CPR and MV in the hospital stay appeared to have higher 1-month and 6-month mortality rates than their male counterparts even after adjustment of age, several critical clinical conditions and the features of healthcare organizations (Table 6). The literature has shown examples where women tended to receive less intensive care and fewer life-supporting treatments, both in the western and the oriental societies [34, 35]. A Canadian study also found that women had a higher death rate after critical illness than men among patients aged 50 or older [34]. However, reasons for such gender differences remain unanswered in these studies. Our analysis is unable to determine the causes of gender difference emerging in the data. The data for our study are also unable to show whether there was overuse or underuse of intensive care among OHCA patients, as well as gender disparity associated with the issue. It certainly calls for more research to clarify these questions.

The final issue we would like to address pertains to the apparent fluctuating pattern in OHCA incidence rates and mortality rates in Taiwan over the period from the early 2000s to the early 2010s. As mentioned previously, changes in policies, regulations and environments may substantially influence the patterns of time-series data. Our data indicate that a higher OHCA incidence rate was generally associated with a higher OHCA mortality, implying that there might be more very fragile OHCA patients delivered to hospitals when the incidence rate was higher. In Taiwan, such a pattern might be related to an NHI policy that expanded provision of prolonged mechanical ventilation care in order to make intensive-care-unit (ICU) care more available in the early 2000s, as well as continuous efforts to promote advanced care planning and appropriate end-of-life care in Taiwan over the past decade [14, 36]. The interaction of the two kinds of events might be a major force that shaped such trends in OHCA incidence rates and mortality rates. Regarding the long-term declining trend in 1-month and 6-month mortality rates, advances in post-resuscitation care might also be a main reason. For both incidence and mortality rates, a significant second-order autoregressive effect emerged, and the direction of effect was negative. The phenomenon suggests that events or shocks occurred two years ago could have influences on the incidence rate and the mortality rate of OHCA in the current year, and the influences might reflect adjustment of previous over-reaction corresponding to the events or shocks.

Our study provides reliable estimates of OHCA incidence rates and mortality rates in Taiwan, because our sample covered nearly all residents in Taiwan and our data contain rich information on individual disease diagnosis, healthcare use, and times of using care in healthcare organizations, as well as the features of healthcare organizations providing care. On the other hand, our study had several limitations. First, we were unable to evaluate the cause of OHCA, pre-hospital care, and the process of resuscitation. All these factors are important for analyzing immediate post-resuscitation outcomes. Second, we were also unable to examine the effects of post-resuscitation care services that may be beneficial for long-term outcomes among OHCA patients with ROSC, such as therapeutic hypothermia and use of neuromuscular blockade agents. Third, we only used mortality as a measurement of outcome, and provided no investigation of the neurological outcome among OHCA survivors, which is a major concern in post-OHCA treatment decisions. This is because we did not have adequate information in the data files available for this present study. Future work is necessary for generating more insightful messages for improving efficacy of care for OHCA and facilitating discussion on post-OHCA treatment.

## Conclusions

The overall incidence rate during the 13 years was 51.1 per 100,000 persons, and the secular trend indicates a sharp increase in the first half of 2000s and then had a declining trend until the early 2010s. The trend in mortality was also curvilinear, revealing a substantial increase in the early 2000s, a subsequent steep decline and finally a modest increase. Both the 30-day and 180-day mortality rates had a long-term decreasing trend over the period (*p*<0.01). For both incidence and mortality rates, a significant second-order autoregressive effect emerged. This suggests that events occurred two years ago could have influences on the incidence rate and the mortality rate of OHCA in the current year. Among OHCA patients requiring MV during the hospital stay, those delivered to regional hospitals and those residing in non-metropolitan areas tended to have higher mortality, suggesting a need for effort to further standardize and improve in-hospital care across all hospitals and to advance pre-hospital care in non-metropolitan areas.

## Supporting Information

S1 Fig Total numbers of incidents with out-of-hospital cardiac arrest and incident rates from 2000 to 2012, for adults aged 18–64. (TIF)

S2 Fig Total numbers of incidents with out-of-hospital cardiac arrest and incident rates from 2000 to 2012, for adults aged 65–74. (TIF)

S3 Fig Total numbers of incidents with out-of-hospital cardiac arrest and incident rates from 2000 to 2012, for adults aged 75–84. (TIF)

S4 Fig Total numbers of incidents with out-of-hospital cardiac arrest and incident rates from 2000 to 2012, for adults aged 85 or older. (TIF)

S5 Fig 1-day mortality rates of out-of-hospital cardiac arrest from 2000 to 2012, by age. (TIF)

S6 Fig 30-day mortality rates of out-of-hospital cardiac arrest from 2000 to 2012, by age. (TIF)

S7 Fig 180-day mortality rates of out-of-hospital cardiac arrest from 2000 to 2012, by age. (TIF)

S1 Table The proportion of hospitals providing care in the National Health Insurance (NHI) program in Taiwan. (DOC)

S2 Table Operational definitions of variables related to organ dysfunctions and diabetes. (DOC)

S3 Table Linear and polynomial regression models of annual OHCA incidence rates (the number per 100,000 persons), for national data of Taiwan from 2000 to 2012, by gender. (DOC)

S4 Table Linear and polynomial regression models of annual OHCA incidence rates (the number per 100,000 persons), for national data of Taiwan from 2000 to 2012, by age. (DOC)

S5 Table Linear and polynomial regression specifications that model the annual OHCA incidence rate (the number per 100,000 persons) in terms of a linear combination of the time period (t) as well as an autoregressive (AR) disturbance process, for national data of Taiwan from 2000 to 2012, by gender. (DOC)

S6 Table Linear and polynomial regression specifications that model the annual OHCA incidence rate (the number per 100,000 persons) in terms of a linear combination of the time period (t) as well as an autoregressive (AR) disturbance process, for national data of Taiwan from 2000 to 2012, by age. (DOC)

S7 Table Linear and polynomial regression models of annual mortality rates (%) among OHCA patients, for national data of Taiwan from 2000 to 2012, by gender. (DOC)

S8 Table Linear and polynomial regression models of annual mortality rates (%) among OHCA patients, for national data of Taiwan from 2000 to 2012, by age. (DOC)

S9 Table Linear and polynomial regression specifications that model the annual mortality rates (%) among OHCA patients in terms of a linear combination of the time period (t) as well as an autoregressive (AR) disturbance process, for national data of Taiwan from 2000 to 2012, by gender. (DOC)

S10 Table Linear and polynomial regression specifications that model the annual mortality rates (%) among OHCA patients in terms of a linear combination of the time period (t) as well as an autoregressive (AR) disturbance process, for national data of Taiwan from 2000 to 2012, by age. (DOC)
