# Supplementary figures and images for: The Secular Trends in the Incidence Rate and Outcomes of Out-of-Hospital Cardiac Arrest in Taiwan—A Nationwide Population-Based Study
Source: PLoS One. 2015 Apr 15;10(4):e0122675. doi: 10.1371/journal.pone.0122675 (PMC4398054; doi:10.1371/journal.pone.0122675)

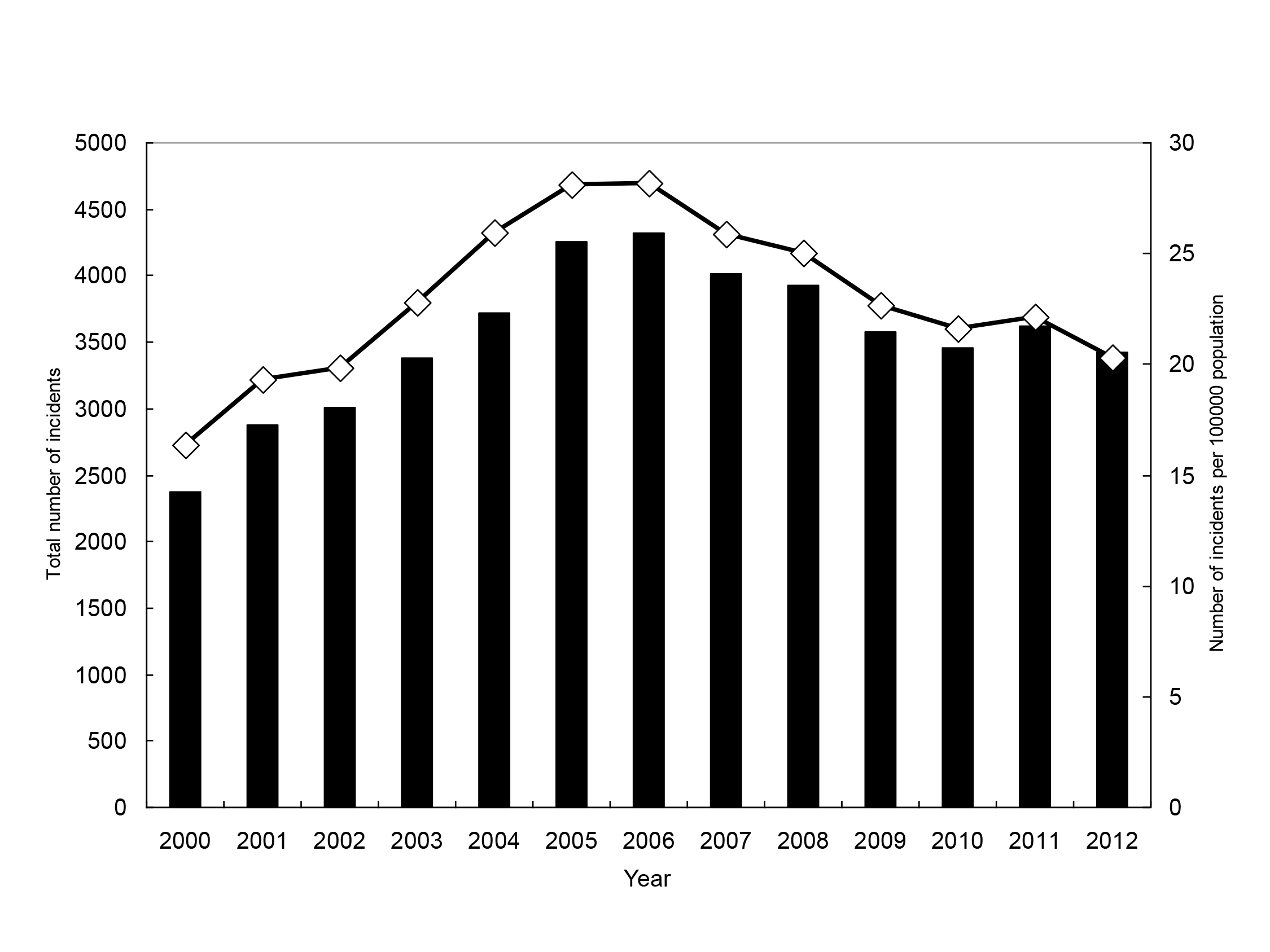

Supplement: S1 Fig — (TIF) [file pone.0122675.s001.tif]

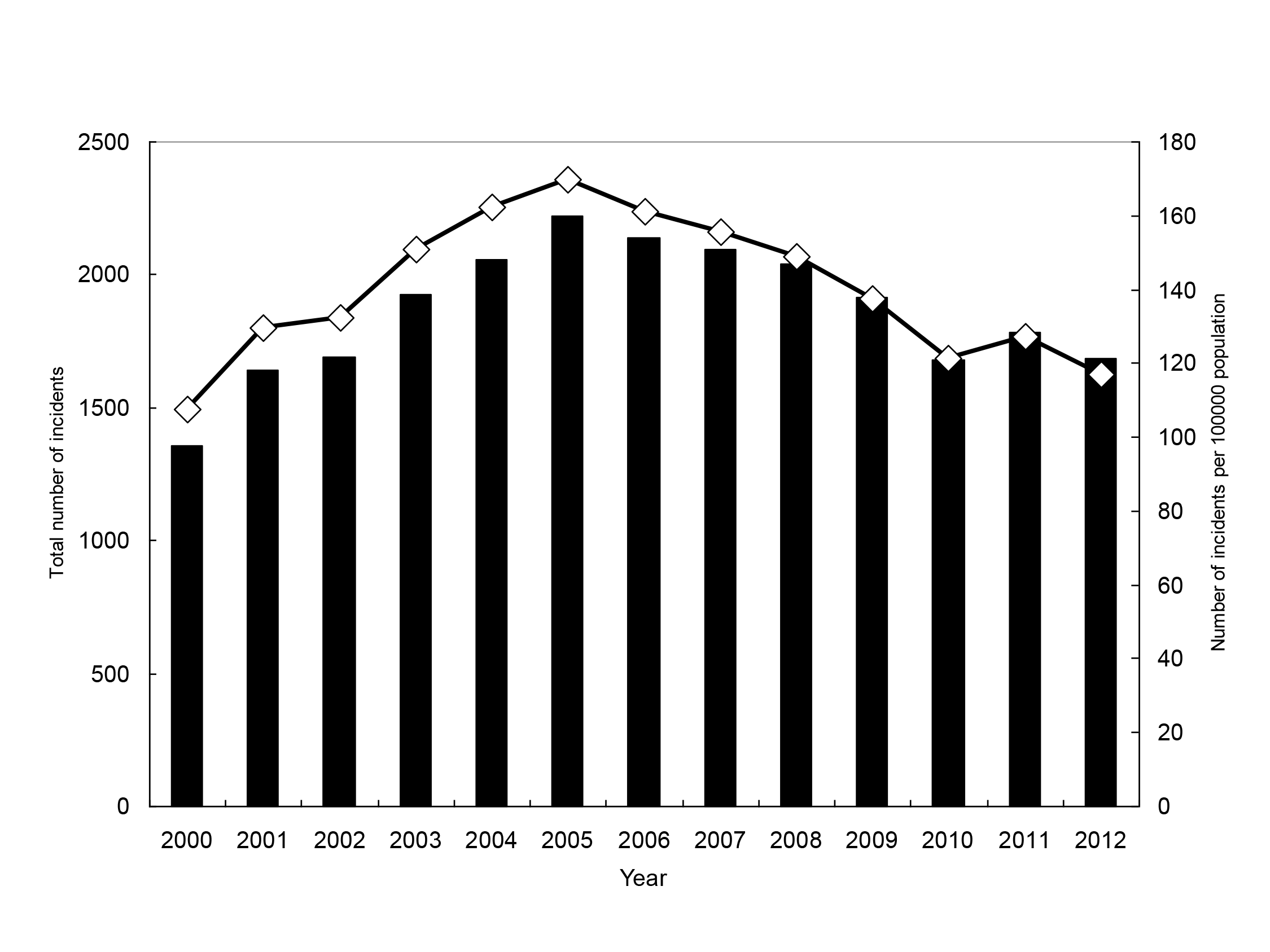

Supplement: S2 Fig — (TIF) [file pone.0122675.s002.tif]

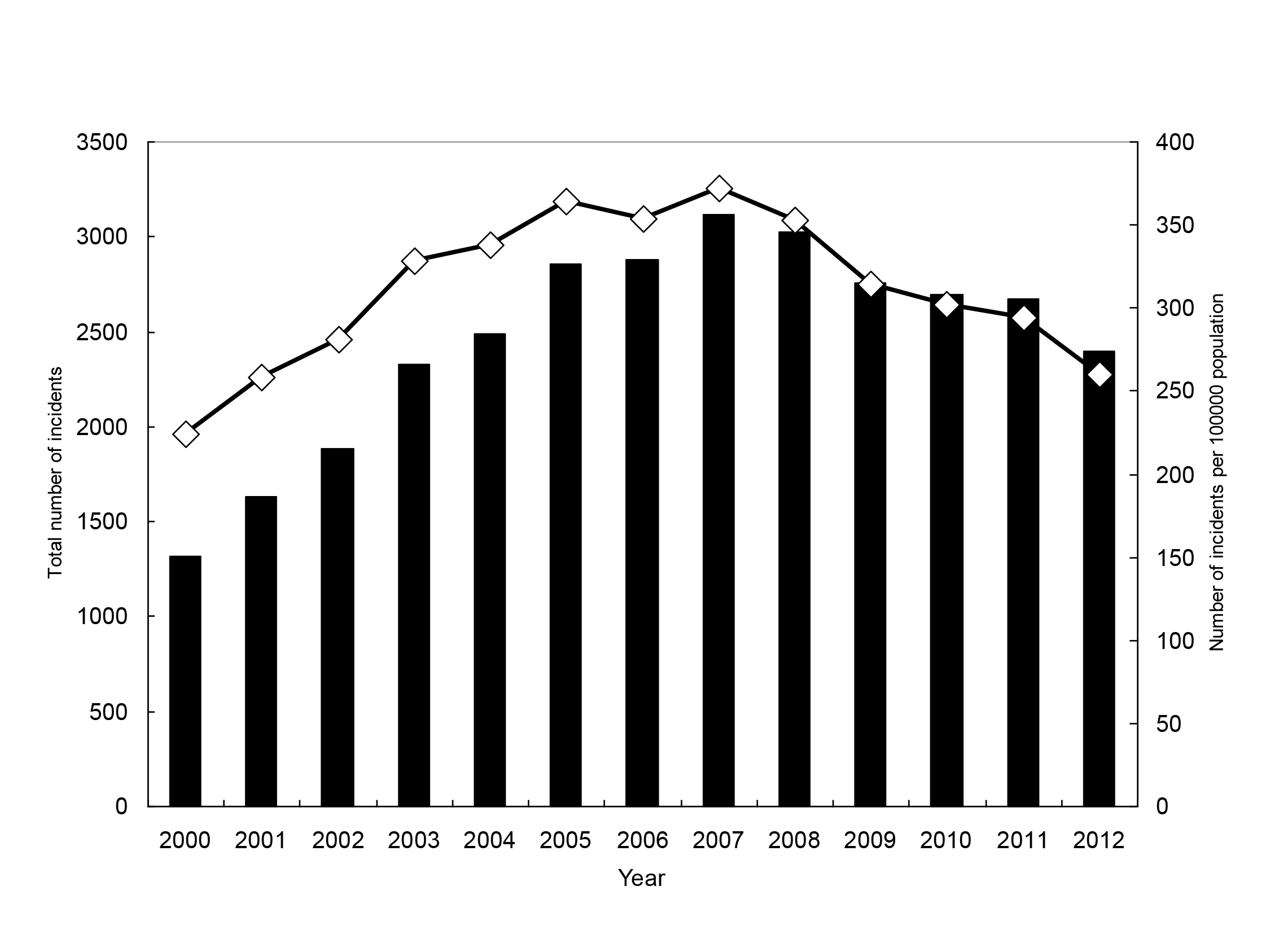

Supplement: S3 Fig — (TIF) [file pone.0122675.s003.tif]

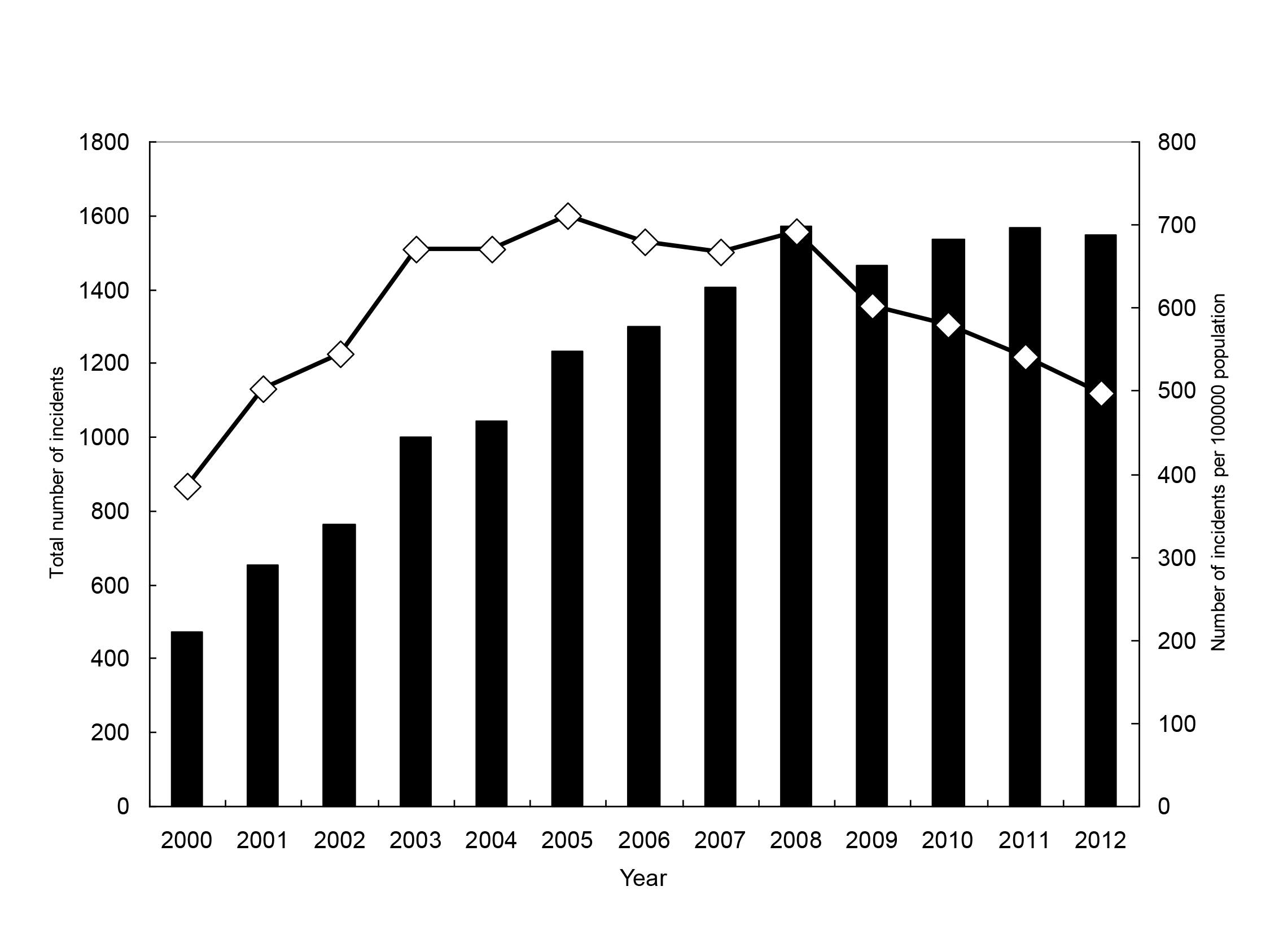

Supplement: S4 Fig — (TIF) [file pone.0122675.s004.tif]

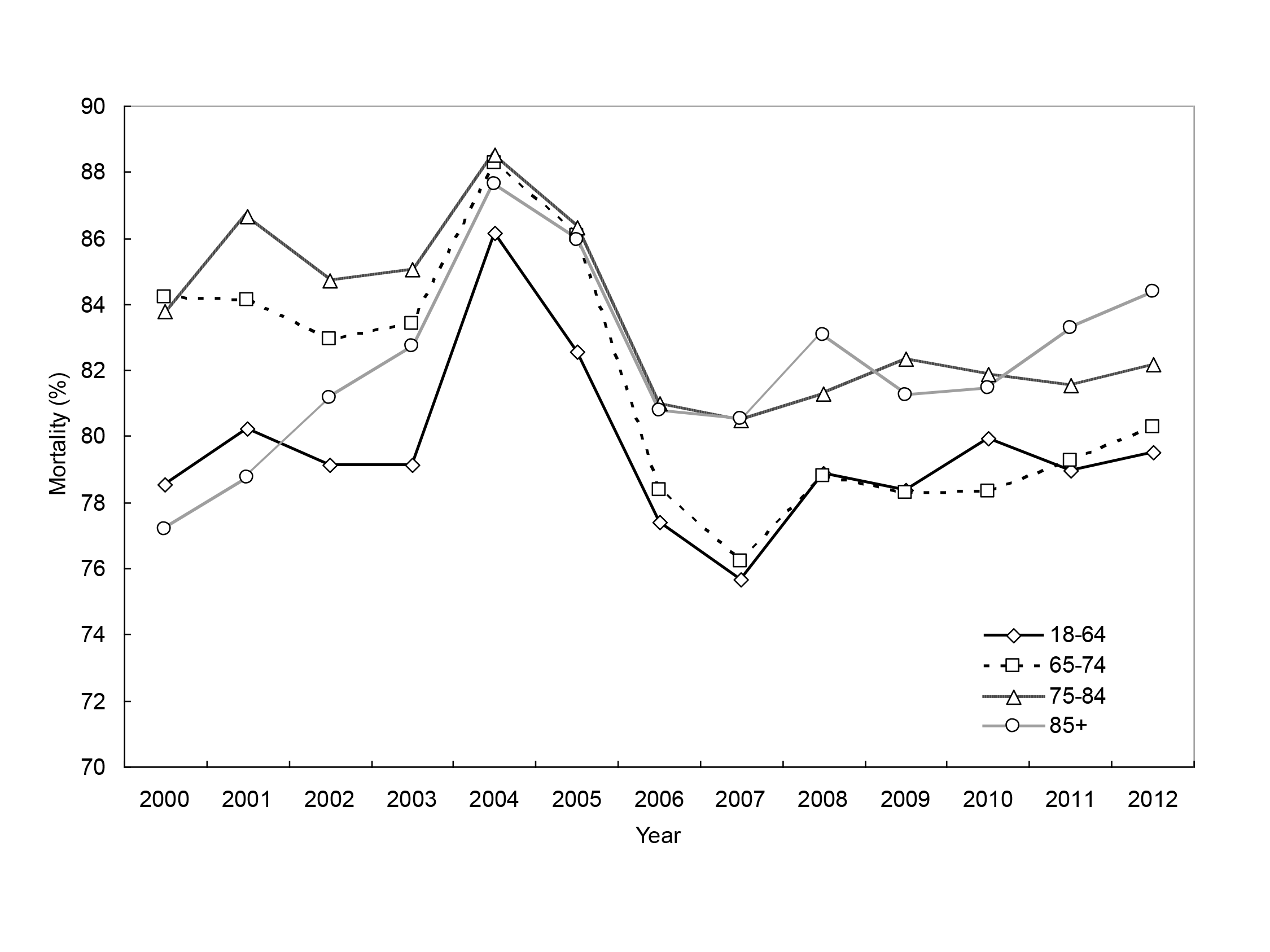

Supplement: S5 Fig — (TIF) [file pone.0122675.s005.tif]

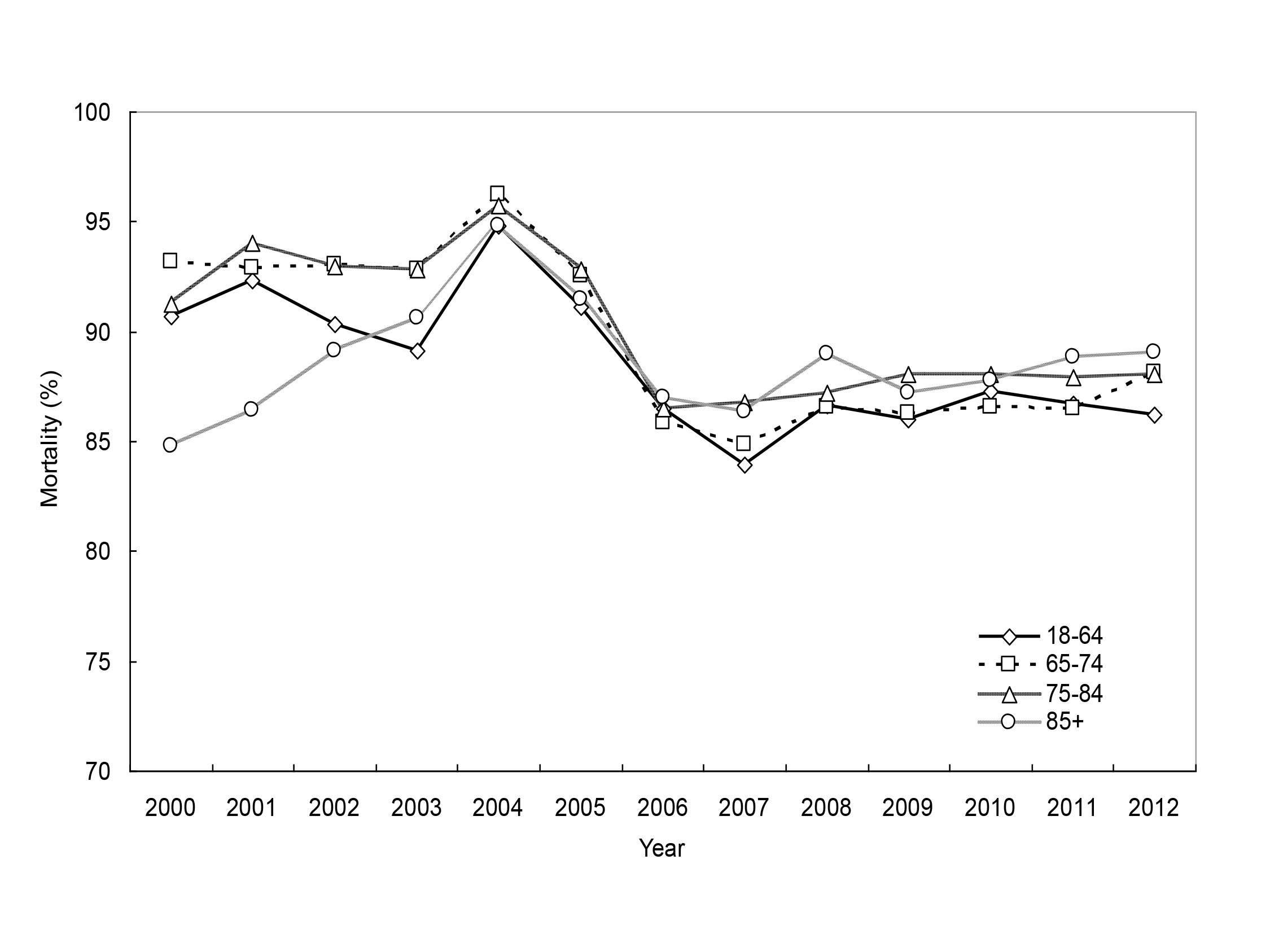

Supplement: S6 Fig — (TIF) [file pone.0122675.s006.tif]

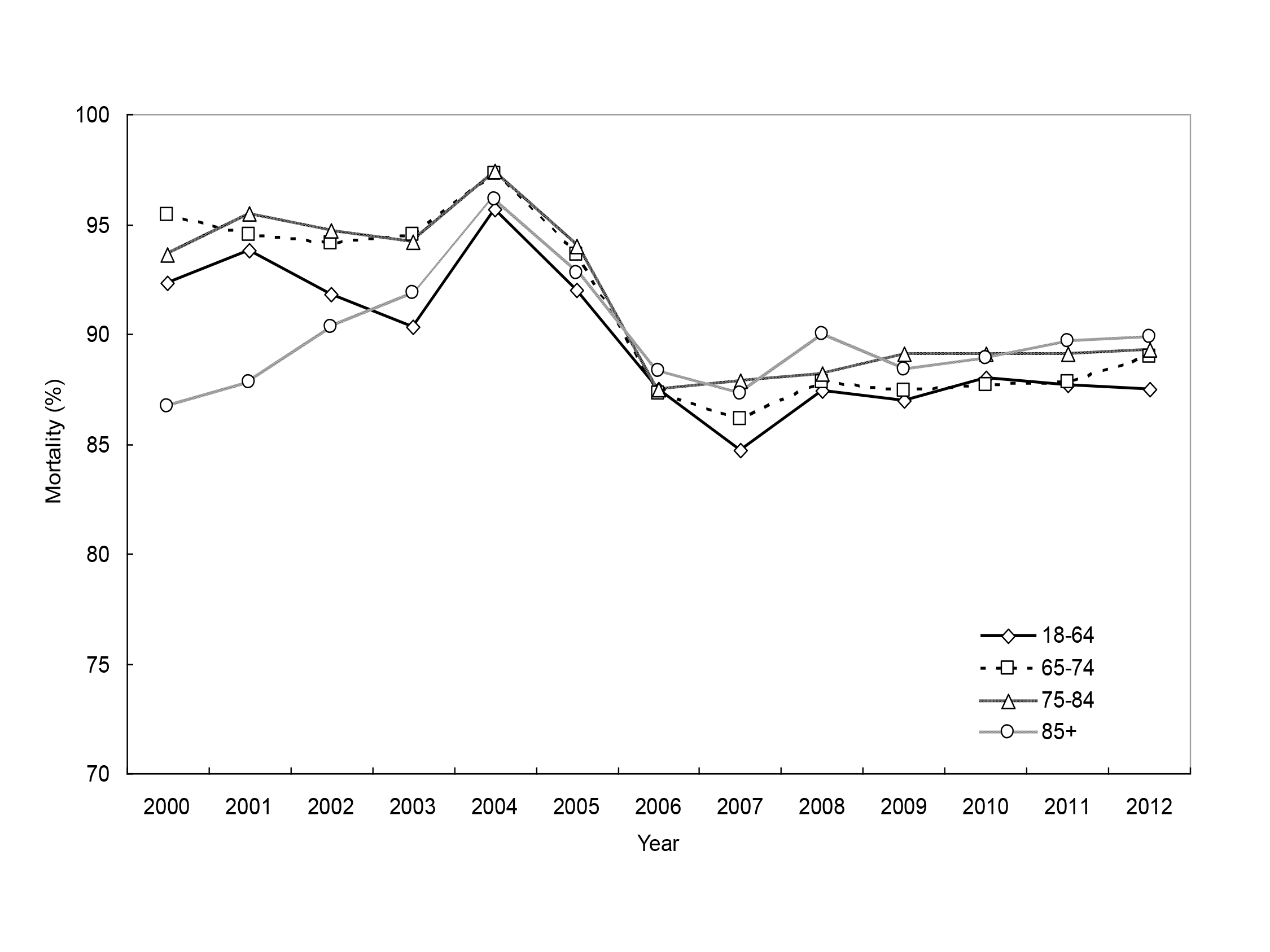

Supplement: S7 Fig — (TIF) [file pone.0122675.s007.tif]
