# Supplementary material for: The Secular Trends in the Incidence Rate and Outcomes of Out-of-Hospital Cardiac Arrest in Taiwan—A Nationwide Population-Based Study
Source: PLoS One. 2015 Apr 15;10(4):e0122675. doi: 10.1371/journal.pone.0122675 (PMC4398054; doi:10.1371/journal.pone.0122675)
Supplement: S1 Table — (DOC) [file pone.0122675.s008.doc]

**S1 Table. The proportion of hospitals providing care in the National Health Insurance (NHI) program in Taiwan.**

| Year | The number of hospitals  in Taiwan | The number of hospitals providing care in the NHI | The proportion of hospitals providing care in the NHI |
| --- | --- | --- | --- |
| 2000 | 617 | 577 | 93.5% |
| 2001 | 593 | 565 | 95.3% |
| 2002 | 574 | 553 | 96.3% |
| 2003 | 558 | 540 | 96.8% |
| 2004 | 556 | 531 | 95.5% |
| 2005 | 531 | 511 | 96.2% |
| 2006 | 523 | 508 | 97.1% |
| 2007 | 507 | 492 | 97.0% |
| 2008 | 493 | 483 | 98.0% |
| 2009 | 496 | 486 | 98.0% |
| 2010 | 492 | 482 | 98.0% |
| 2011 | 491 | 479 | 97.6% |
| 2012 | 488 | 478 | 98.0% |

Data source: The Ministry of Health and Welfare, Taiwan. (Available at:

<http://www.mohw.gov.tw/cht/DOS/Statistic.aspx?f_list_no=312&fod_list_no=5048> and

<http://www.mohw.gov.tw/cht/DOS/Statistic.aspx?f_list_no=312&fod_list_no=1828> )
