# Supplementary material for: The Secular Trends in the Incidence Rate and Outcomes of Out-of-Hospital Cardiac Arrest in Taiwan—A Nationwide Population-Based Study
Source: PLoS One. 2015 Apr 15;10(4):e0122675. doi: 10.1371/journal.pone.0122675 (PMC4398054; doi:10.1371/journal.pone.0122675)
Supplement: S2 Table — (DOC) [file pone.0122675.s009.doc]

**S2 Table. Operational definitions of variables related to organ dysfunctions and diabetes.**

| Organ dysfunction/disease |  | Operational definition |
| --- | --- | --- |
| ***Organ dysfunction*** |  |  |
| Heart failure |  |  |
| Prior to the index admission |  | Since January 1, 1997 until the index admission date, there were at least two times of admissions or visits for emergency care related to at least one of the following diagnoses: 402.01, 402.11, 402.91, 404.01, 404.03, 404.11, 404.13, 404.91, 404.93, 428.00, 428.01, 428.09, 428.20, 428.21, 428.22, 428.23, 428.30, 428.31, 428.32, 428.33, 428.40, 428.41, 428.42, and 428.43.  (Refer to: Bonow RO, Bennett S, Casey DE Jr *et al.* ACC/AHA Clinical Performance Measures for Adults With Chronic Heart Failure: A Report of the American College of Cardiology/American Heart Association Task Force on Performance Measures (Writing Committee to Develop Heart Failure Clinical Performance Measures): Endorsed by the Heart Failure Society of America. *Circulation* 2005; **112:** 1853-1887.) |
| Renal failure |  |  |
| Prior to the index admission |  | At the time of index admission, the patient had **a valid benefits card through a catastrophic illness registry system** in the National Health Insurance due to his or her need for dialysis. |
| Hepatic failure |  |  |
| Prior to the index admission |  | Since January 1, 1997 until the index admission date, there was at least one application for acquiring **a benefits card through a catastrophic illness registry system** in the National Health Insurance for at least one of the following diagnoses: 571.2, 571.5, and 571.6. Additionally, there was no liver transplantation between the day of the last application of NHI benefits for catastrophic illness and the index admission date. |
| Stroke |  |  |
| Prior to the index admission |  | Since January 1, 1997 until the index admission date, there was care use in **the intensive care unit** for at least one of the following diagnoses: 430, 431, 432, 433, 434, 435, 436, and 437. |
| Severe obstructive airway diseases | |  |
| Prior to the index admission |  | In the one year prior the index admission date, there were at least two times of admissions or visits for emergency care related to at least one of the following diagnoses: 490-496. Additionally, during the one year, **the medication possession ratio for bronchodilators prescribed in outpatient settings had to be > 80%** (i.e., bronchodilators were prescribed for at least 365*0.8 = 292 days). |
| ***Diabetes mellitus prior to the index admission*** | | |
| Insulin |  | In the three months prior the index admission date, there was at least one prescription of insulin. |
| Oral hypoglycemic agent |  | In the three months prior the index admission date, there was at least one prescription of oral hypoglycemic agent (for diabetic patients using no insulin). |
