# Supplementary material for: The Secular Trends in the Incidence Rate and Outcomes of Out-of-Hospital Cardiac Arrest in Taiwan—A Nationwide Population-Based Study
Source: PLoS One. 2015 Apr 15;10(4):e0122675. doi: 10.1371/journal.pone.0122675 (PMC4398054; doi:10.1371/journal.pone.0122675)
Supplement: S3 Table — (DOC) [file pone.0122675.s010.doc]

**S3 Table. Linear and polynomial regression models of annual OHCA incidence rates (the number per 100,000 persons), for national data of Taiwan from 2000 to 2012, by gender.**

|  | Both genders | | | |  | Men | | | | |  | Women | | | | |
| --- | --- | --- | --- | --- | --- | --- | --- | --- | --- | --- | --- | --- | --- | --- | --- | --- |
|  | Coefficient | | 95% CI | |  | Coefficient | | | 95% CI | |  | Coefficient | | | 95% CI | |
| Simple linear regression models with robust variance estimates | | | | | |  | | |  |  |  |  | | |  |  |
| Intercept | 45.33 | *** | (34.97－ | 55.69) |  | 59.15 | | *** | (45.03－ | 73.26) |  | 31.73 | | *** | (24.75－ | 38.71) |
| t | 0.94 |  | (-0.48－ | 2.35) |  | 1.20 | |  | (-0.70－ | 3.10) |  | 0.74 | |  | (-0.24－ | 1.72) |
|  | R^2^=0.1884 | |  |  |  | R^2^=0.1727 | | |  |  |  | R^2^=0.2325 | | |  |  |
| Polynomial models with the quadratic term of “t” and with robust variance estimates | | | | | | | | | | |  |  | | |  |  |
| Intercept | 32.97 | *** | (30.51－ | 35.43) |  | 42.55 | *** | | (39.12－ | 45.98) |  | 23.18 | *** | | (21.16－ | 25.19) |
| t | 7.68 | *** | (6.62－ | 8.74) |  | 10.26 | *** | | (8.64－ | 11.87) |  | 5.41 | *** | | (4.62－ | 6.19) |
| t^2^ | -0.56 | *** | (-0.65－ | -0.47) |  | -0.75 | *** | | (-0.90－ | -0.61) |  | -0.39 | *** | | (-0.45－ | -0.33) |
|  | **R^2^=0.9326** | |  |  |  | **R^2^=0.9199** | | |  |  |  | **R^2^=0.9357** | | |  |  |
| Polynomial models with the quadratic term and the cubic term of “t” and with robust variance estimates | | | | | | | | | | |  |  | | |  |  |
| Intercept | 31.67 | *** | (27.60－ | 35.73) |  | 40.52 | | *** | (34.76－ | 46.27) |  | 22.62 | | *** | (19.60－ | 25.63) |
| t | 9.32 | *** | (6.05－ | 12.60) |  | 12.81 | | *** | (8.10－ | 17.52) |  | 6.11 | | *** | (3.78－ | 8.45) |
| t^2^ | -0.92 | * | (-1.59－ | -0.25) |  | -1.31 | | * | (-2.24－ | -0.37) |  | -0.54 | | * | (-1.04－ | -0.04) |
| t^3^ | 0.02 |  | (-0.02－ | 0.06) |  | 0.03 | |  | (-0.02－ | 0.08) |  | 0.01 | |  | (-0.02－ | 0.04) |
|  | **R^2^=0.9421** | |  |  |  | **R^2^=0.9326** | | |  |  |  | **R^2^=0.9392** | | |  |  |

^*^ p<0.05; ^**^p<0.01; ^***^p<0.001.

Abbreviations: CI, confidence interval; OHCA, out-of-hospital cardiac arrest.

^a^For the year 2000, t=0; t=1 for the year 2001, t=2 for the year 2002, and so on.
