# Supplementary material for: The Secular Trends in the Incidence Rate and Outcomes of Out-of-Hospital Cardiac Arrest in Taiwan—A Nationwide Population-Based Study
Source: PLoS One. 2015 Apr 15;10(4):e0122675. doi: 10.1371/journal.pone.0122675 (PMC4398054; doi:10.1371/journal.pone.0122675)
Supplement: S4 Table — (DOC) [file pone.0122675.s011.doc]

**S4 Table. Linear and polynomial regression models of annual OHCA incidence rates (the number per 100,000 persons), for national data of Taiwan from 2000 to 2012, by age.**

|  | Aged 18~64 | | | | |  | Aged 65~74 | | | | |  | Aged 75~84 | | | | |  | | Aged 85+ | | | | |
| --- | --- | --- | --- | --- | --- | --- | --- | --- | --- | --- | --- | --- | --- | --- | --- | --- | --- | --- | --- | --- | --- | --- | --- | --- |
|  | Coefficient | | | 95%CI | |  | Coefficient | | | 95%CI | |  | Coefficient | | 95%CI | | |  | | Coefficient | | 95%CI | | |
| Simple linear regression models with robust variance estimates | | | | | | | | | | | | | | | | | |  |  | |  |  |  |  |
| Intercept | 21.60 | *** | (17.01－ | | 26.20) |  | 142.8 | *** | (114.52－ | | 171.08) |  | 295.25 | *** | | (233.68－ | 356.82) |  | 569.19 | | *** | (421.95－ | 716.42) |  |
| t | 0.22 |  | (-0.38－ | | 0.81) |  | -0.46 |  | (-4.09－ | | 3.18) |  | 2.61 |  | | (-6.11－ | 11.32) |  | 4.35 | |  | (-15.31－ | 24.01) |  |
|  | R^2^=0.0580 | |  | |  |  | R^2^=0.0084 | |  | |  |  | R^2^=0.0488 | | |  |  |  | R^2^=0.0301 | | |  |  |  |
| Polynomial models with the quadratic term of “t” and with robust variance estimates | | | | | | | | | | | | | | | | | |  |  | |  |  |  |  |
| Intercept | 16.25 | *** | (14.91－ | | 17.59) |  | 112.39 | *** | (103.71－ | | 121.06) |  | 221.05 | *** | | (209.96－ | 232.14) |  | 412.28 | | *** | (367.83－ | 456.73) |  |
| t | 3.14 | *** | (2.37－3.91 | | 3.91) |  | 16.13 | *** | (11.87－ | | 20.40) |  | 43.08 | *** | | (37.67－ | 48.49) |  | 89.93 | | *** | (72.38－ | 107.48) |  |
| t^2^ | -0.24 | *** | (-0.31－ | | -0.17) |  | -1.38 | *** | (-1.77－ | | -0.99) |  | -3.37 | *** | | (-3.81－ | -2.94) |  | -7.13 | | *** | (-8.59－ | -5.68) |  |
|  | **R^2^=0.8404** | |  | |  |  | **R^2^=0.8508** | |  | |  |  | **R^2^=0.9464** | | |  |  |  | **R^2^=0.9206** | | |  |  |  |
| Polynomial models with the quadratic term and the cubic term of “t” and with robust variance estimates | | | | | | | | | | | | | | | | | |  |  | |  |  |  |  |
| Intercept | 15.31 | *** | (12.89－ | | 17.73) |  | 104.22 | *** | (95.38－ | | 113.06) |  | 216.33 | *** | | (199.18－ | 233.48) |  | 381.62 | | *** | (364.23－ | 399.00) |  |
| t | 4.32 | ** | (2.25－ | | 6.39) |  | 26.41 | *** | (18.38－ | | 34.43) |  | 49.01 | *** | | (34.53－ | 63.50) |  | 128.50 | | *** | (102.92－ | 154.08) |  |
| t^2^ | -0.50 | * | (-0.91－ | | -0.09) |  | -3.61 | ** | (-5.29－ | | -1.93) |  | -4.66 | *** | | (-7.83－ | -1.49) |  | -15.50 | | *** | (-21.69－ | -9.30) |  |
| t^3^ | 0.01 |  | (-0.01－ | | 0.04) |  | 0.12 | * | (0.04－ | | 0.21) |  | 0.07 |  | | (-0.11－ | 0.25) |  | 0.46 | |  | (0.11－ | 0.82) |  |
|  | **R^2^=0.8679** | |  | |  |  | **R^2^=0.9203** | |  | |  |  | **R^2^=0.9505** | | |  |  |  | **R^2^=0.9595** | | |  |  |  |

^*^ p<0.05; ^**^p<0.01; ^***^p<0.001.

Abbreviations: CI, confidence interval; OHCA, out-of-hospital cardiac arrest.

^a^For the year 2000, t=0; t=1 for the year 2001, t=2 for the year 2002, and so on.
