# Supplementary material for: The Secular Trends in the Incidence Rate and Outcomes of Out-of-Hospital Cardiac Arrest in Taiwan—A Nationwide Population-Based Study
Source: PLoS One. 2015 Apr 15;10(4):e0122675. doi: 10.1371/journal.pone.0122675 (PMC4398054; doi:10.1371/journal.pone.0122675)
Supplement: S5 Table — (DOC) [file pone.0122675.s012.doc]

**S5 Table. Linear and polynomial regression specifications that model the annual OHCA incidence rate (the number per 100,000 persons) in terms of a linear combination of the time period (t) as well as an autoregressive (AR) disturbance process, for national data of Taiwan from 2000 to 2012, by gender.**

|  | Both genders | | | |  | Men | | | | |  | Women | | | | |
| --- | --- | --- | --- | --- | --- | --- | --- | --- | --- | --- | --- | --- | --- | --- | --- | --- |
|  | Coefficient | | 95% CI | |  | Coefficient | | | 95% CI | |  | Coefficient | | | 95% CI | |
| Simple linear specifications with robust variance estimates | | | | | |  | | |  |  |  |  | | |  |  |
| Intercept | 44.26 | *** | (32.46－ | 56.06) |  | 58.20 | | *** | (41.84－ | 74.56) |  | 31.02 | | *** | (20.34－ | 41.70) |
| t | 0.92 |  | (-0.75－ | 2.59) |  | 1.09 | |  | (-1.20－ | 3.38) |  | 0.72 | |  | (-0.57－ | 2.02) |
| Lag of 1 | 0.91 | *** | (0.49－ | 1.34) |  | 0.91 | | *** | (0.46－ | 1.36) |  | 0.99 | | *** | (0.58－ | 1.39) |
| Lag of 2 | 0.45 |  | (-0.16－ | 1.06) |  | 0.45 | |  | (-0.21－ | 1.11) |  | 0.21 | |  | (-0.42－ | 0.84) |
| Lag of 3 | -0.73 | ** | (-1.14－ | -0.31) |  | -0.72 | | *** | (-1.08－ | -0.36) |  | -0.55 | | * | (-1.07－ | -0.02) |
| Sigma^b^ | 2.39 | *** | (1.62－ | 3.16) |  | 3.34 | | *** | (2.31－ | 4.37) |  | 2.06 | | *** | (1.47－ | 2.65) |
| Polynomial specifications with the quadratic term of “t” and with robust variance estimates | | | | | | | | | | |  |  | | |  |  |
| Intercept | 32.89 | *** | (29.60－ | 36.18) |  | 43.02 | *** | | (39.94－ | 46.11) |  | 22.41 | *** | | (20.97－ | 23.84) |
| t | 7.93 | *** | (6.64－ | 9.22) |  | 10.42 | *** | | (9.09－ | 11.75) |  | 5.86 | *** | | (5.24－ | 6.47) |
| t^2^ | -0.59 | *** | (-0.69－ | -0.49) |  | -0.78 | *** | | (-0.88－ | -0.68) |  | -0.43 | *** | | (-0.48－ | -0.38) |
| Lag of 1 | 0.27 |  | (-0.19－ | 0.72) |  | 0.28 |  | | (-0.23－ | 0.78) |  | -0.13 |  | | (-0.66－ | 0.39) |
| Lag of 2 | -0.27 |  | (-0.84－ | 0.30) |  | -0.11 |  | | (-0.77－ | 0.55) |  | -0.63 | * | | (-1.12－ | -0.14) |
| Lag of 3 | -0.51 |  | (-1.07－ | 0.06) |  | -0.66 |  | | (-1.34－ | 0.03) |  | -0.41 |  | | (-0.82－ | 0.01) |
| Sigma^b^ | 1.48 | *** | (0.94－ | 2.03) |  | 1.88 | *** | | (1.19－ | 2.56) |  | **1.10** | *** | | (0.78－ | 1.42) |
| **Polynomial specifications with the *quadratic* term and the *cubic* term of “t” and with robust variance estimates** | | | | | | | | | | |  |  | | |  |  |
| Intercept | 30.48 | *** | (23.02－ | 37.94) |  | 39.25 | | *** | (28.44－ | 50.06) |  | 21.63 | | *** | (19.33－ | 23.92) |
| t | 9.94 | *** | (5.22－ | 14.66) |  | 13.55 | | *** | (6.72－ | 20.38) |  | 6.52 | | *** | (4.84－ | 8.21) |
| t^2^ | -0.97 | * | (-1.76－ | -0.18) |  | -1.38 | | * | (-2.51－ | -0.24) |  | -0.56 | | *** | (-0.87－ | -0.25) |
| t^3^ | 0.02 |  | (-0.02－ | 0.06) |  | 0.03 | |  | (-0.02－ | 0.09) |  | 0.01 | |  | (-0.01－ | 0.02) |
| Lag of 1 | 0.15 |  | (-0.28－ | 0.59) |  | 0.19 | |  | (-0.24－ | 0.62) |  | -0.21 | |  | (-0.75－ | 0.33) |
| Lag of 2 | -0.51 |  | (-1.71－ | 0.69) |  | -0.39 | |  | (-1.56－ | 0.78) |  | -0.70 | | * | (-1.25－ | -1.43) |
| Lag of 3 | -0.40 |  | (-1.12－ | 0.31) |  | -0.54 | |  | (-1.32－ | 0.23) |  | -0.38 | |  | (-0.81－ | 0.06) |
| Sigma^b^ | 1.37 | *** | (1.06－ | 1.69) |  | **1.74** | | *** | (0.29－ | 2.18) |  | **1.06** | | *** | (0.71－ | 1.40) |

^*^ p<0.05; ^**^p<0.01; ^***^p<0.001.

Abbreviations: CI, confidence interval; OHCA, out-of-hospital cardiac arrest.

^a^For the year 2000, t=0; t=1 for the year 2001, t=2 for the year 2002, and so on. The models include lags of 1, 2 and 3 of the structural disturbance.

^b^Sigma is ***the estimated standard deviation of the white-noise disturbance.***
