# Supplementary material for: The Secular Trends in the Incidence Rate and Outcomes of Out-of-Hospital Cardiac Arrest in Taiwan—A Nationwide Population-Based Study
Source: PLoS One. 2015 Apr 15;10(4):e0122675. doi: 10.1371/journal.pone.0122675 (PMC4398054; doi:10.1371/journal.pone.0122675)
Supplement: S6 Table — (DOC) [file pone.0122675.s013.doc]

**S6 Table. Linear and polynomial regression specifications that model the annual OHCA incidence rate (the number per 100,000 persons) in terms of a linear combination of the time period (t) as well as an autoregressive (AR) disturbance process, for national data of Taiwan from 2000 to 2012, by age.**

|  | Aged 18~64 | | | | |  | Aged 65~74 | | | | |  | Aged 75~84 | | | | |  | | Aged 85+ | | | | |
| --- | --- | --- | --- | --- | --- | --- | --- | --- | --- | --- | --- | --- | --- | --- | --- | --- | --- | --- | --- | --- | --- | --- | --- | --- |
|  | Coefficient | | | 95%CI | |  | Coefficient | | | 95%CI | |  | Coefficient | | 95%CI | | |  | | Coefficient | | 95%CI | | |
| Simple linear specifications with robust variance estimates | | | | | | | | | | | | | | | | | |  |  | |  |  |  |  |
| Intercept | 21.16 | *** | (15.46－ | | 26.9) |  | 133.26 | *** | (101.18－ | | 165.33) |  | 279.31 | *** | | (183.85－ | 374.76) |  | 487.14 | | *** | (257.59－ | 716.69) |  |
| t | 0.25 |  | (-0.50－ | | 1.00) |  | 0.93 |  | (-3.20－ | | 5.07) |  | 2.38 |  | | (-10.46－ | 15.23) |  | 10.54 | |  | (-14.81－ | 35.89) |  |
| Lag of 1 | 0.92 | *** | (0.42－ | | 1.42) |  | 0.79 | *** | (0.44－ | | 1.14) |  | 1.01 | *** | | (0.73－ | 1.29) |  | 0.94 | | *** | (0.64－ | 1.24) |  |
| Lag of 2 | 0.28 |  | (-0.50－ | | 1.05) |  | 0.58 | ** | (0.20－ | | 0.95) |  | 0.32 |  | | (-0.35－ | 0.99) |  | 0.50 | | ** | (0.17－ | 0.82) |  |
| Lag of 3 | -0.61 | ** | (-0.99－ | | -0.23) |  | -0.79 | *** | (-1.05－ | | -0.54) |  | -0.60 | ** | | (-1.00－ | -0.21) |  | -0.71 | | *** | (-1.08－ | -0.35) |  |
| Sigma^b^ | 1.29 | *** | (0.92－ | | 1.67) |  | 7.15 | *** | (5.16－ | | 9.13) |  | 17.18 | *** | | (12.33－ | 22.04) |  | 37.24 | | *** | (22.18－ | 52.29) |  |
| Polynomial specifications with the quadratic term of “t” and with robust variance estimates | | | | | | | | | | | | | | | | | |  |  | |  |  |  |  |
| Intercept | 16.21 | *** | (14.91－ | | 17.52) |  | 114.08 | *** | (103.19－ | | 124.97) |  | 217.31 | *** | | (210.21－ | 224.41) |  | 422.49 | | *** | (371.22－ | 473.75) |  |
| t | 3.30 | *** | (2.75－ | | 3.85) |  | 15.72 | *** | (10.69－ | | 20.75) |  | 45.63 | *** | | (42.45－ | 48.81) |  | 87.47 | | *** | (71.56－ | 103.38) |  |
| t^2^ | -0.26 | *** | (-0.30－ | | -0.22) |  | -1.36 | *** | (-1.80－ | | -0.91) |  | -3.63 | *** | | (-3.89－ | -3.37) |  | -7.02 | | *** | (-8.14－ | -5.91) |  |
| Lag of 1 | 0.38 |  | (-0.22－ | | 0.98) |  | 0.34 |  | (-0.19－ | | 0.88) |  | -0.36 |  | | (-0.82－ | 0.09) |  | -0.09 | |  | (-0.70－ | 0.52) |  |
| Lag of 2 | -0.17 |  | (-0.92－ | | 0.59) |  | -0.04 |  | (-1.01－ | | 0.93) |  | -0.58 | * | | (-1.03－ | -0.13) |  | 0.02 | |  | (-0.70－ | 0.73) |  |
| Lag of 3 | -0.62 |  | (-1.33－ | | 0.09) |  | -0.47 |  | (-1.20－ | | 0.27) |  | -0.57 | ** | | (-0.96－ | -0.17) |  | -0.41 | |  | (-1.23－ | 0.41) |  |
| Sigma^b^ | 0.73 | *** | (0.48－ | | 0.97) |  | 5.92 | *** | (3.69－ | | 8.15) |  | **7.89** | *** | | (5.30－ | 10.48) |  | 24.07 | | *** | (17.89－ | 30.25) |  |
| **Polynomial specifications with the *quadratic* term and the *cubic* term of “t” and with robust variance estimates** | | | | | | | | | | | | | | | | | |  |  | |  |  |  |  |
| Intercept | 14.73 | *** | (12.04－ | | 17.41) |  | 98.86 | *** | (87.08－ | | 110.63) |  | 211.54 | *** | | (198.54－ | 224.54) |  | 375.34 | | *** | (359.76－ | 390.92) |  |
| t | 4.64 | *** | (2.89－ | | 6.39) |  | 29.40 | *** | (22.08－ | | 36.72) |  | 50.60 | *** | | (40.86－ | 60.33) |  | 132.28 | | *** | (122.60－ | 141.96) |  |
| t^2^ | -0.52 | ** | (-0.83－ | | -0.22) |  | -3.99 | *** | (-5.23－ | | -2.75) |  | -4.62 | *** | | (-6.47－ | -2.77) |  | -16.05 | | *** | (-17.64－ | -14.45) |  |
| t^3^ | 0.01 |  | (-0.001－ | | 0.03) |  | 0.14 |  | (0.08－ | | 0.20) |  | 0.05 |  | | (-0.04－ | 0.15) |  | 0.49 | | *** | (0.41－ | 0.56) |  |
| Lag of 1 | 0.28 |  | (-0.18－ | | 0.73) |  | -0.19 |  | (-0.65－ | | 0.27) |  | -0.47 |  | | (-1.02－ | 0.09) |  | -1.18 | | *** | (-1.64－ | -0.72) |  |
| Lag of 2 | -0.34 |  | (-1.07－ | | 0.40) |  | -0.61 |  | (-1.53－ | | 0.31) |  | -0.66 | ** | | (-1.17－ | -0.15) |  | -0.97 | | *** | (-1.44－ | -0.51) |  |
| Lag of 3 | -0.59 | * | (-1.17－ | | -0.01) |  | -0.45 |  | (-1.07－ | | 0.17) |  | -0.54 | ** | | (-0.95－ | -0.12) |  | -0.47 | | ** | (-0.83－ | -0.12) |  |
| Sigma^b^ | **0.63** | *** | (0.41－ | | 0.85) |  | 4.01 | *** | (2.61－ | | 5.42) |  | **7.56** | *** | | (5.28－ | 9.84) |  | **10.72** | | *** | (5.87－ | 15.58) |  |

^*^ p<0.05; ^**^p<0.01; ^***^p<0.001.

Abbreviations: CI, confidence interval; OHCA, out-of-hospital cardiac arrest.

^a^For the year 2000, t=0; t=1 for the year 2001, t=2 for the year 2002, and so on. The models include lags of 1, 2 and 3 of the structural disturbance.

^b^Sigma is ***the estimated standard deviation of the white-noise disturbance.***
