# Supplementary material for: The Secular Trends in the Incidence Rate and Outcomes of Out-of-Hospital Cardiac Arrest in Taiwan—A Nationwide Population-Based Study
Source: PLoS One. 2015 Apr 15;10(4):e0122675. doi: 10.1371/journal.pone.0122675 (PMC4398054; doi:10.1371/journal.pone.0122675)
Supplement: S7 Table — (DOC) [file pone.0122675.s014.doc]

**S7 Table. Linear and polynomial regression models of annual mortality rates (%) among OHCA patients, for national data of Taiwan from 2000 to 2012, by gender.**

|  | Both genders | | | |  | Men | | | | |  | Women | | | | |
| --- | --- | --- | --- | --- | --- | --- | --- | --- | --- | --- | --- | --- | --- | --- | --- | --- |
|  | Coefficient | | 95% CI | |  | Coefficient | | | 95% CI | |  | Coefficient | | | 95% CI | |
| **1-day mortality** | | | | | | | | | | | | | | | | |
| Simple linear regression models with robust variance estimates | | | | | |  | | |  |  |  |  | | |  |  |
| Intercept | 84.92 | *** | (79.87－ | 89.96) |  | 83.70 | | *** | (81.20－ | 86.21) |  | 81.27 | | *** | (78.81－ | 83.74) |
| t | -0.45 |  | (-1.01－ | 0.10) |  | -0.26 | | * | (-0.49－ | -0.03) |  | -0.19 | |  | (-0.44－ | 0.06) |
|  | R^2^=0.2223 | |  |  |  | R^2^=0.1599 | | |  |  |  | R^2^=0.0908 | | |  |  |
| Polynomial models with the quadratic term of “t” and with robust variance estimates | | | | | | | | | | |  |  | | |  |  |
| Intercept | 84.95 | *** | (77.85－ | 92.06) |  | 83.35 | *** | | (81.11－ | 85.59) |  | 81.36 | *** | | (78.58－ | 84.13) |
| t | -0.47 |  | (-2.72－ | 1.77) |  | -0.06 |  | | (-1.49－ | 1.36) |  | -0.24 | *** | | (-1.79－ | 1.32) |
| t^2^ | 0.001 |  | (-0.16－ | 0.17) |  | -0.02 |  | | (-0.14－ | 0.11) |  | 0.004 | *** | | (-0.13－ | 0.14) |
|  | R^2^=0.2223 | |  |  |  | R^2^=0.1668 | | |  |  |  | R^2^=0.0912 | | |  |  |
| **Polynomial models with the *quadratic* term and the *cubic* term of “t” and with robust variance estimates** | | | | | | | | | | |  |  | | |  |  |
| Intercept | 81.81 | *** | (78.61－ | 85.02) |  | 81.55 | | *** | (79.67－ | 83.43) |  | 79.02 | | *** | (77.22－ | 80.83) |
| t | 3.48 |  | (-0.04－ | 6.99) |  | 2.20 | |  | (-0.95－ | 5.36) |  | 2.70 | | *** | (-0.12－ | 5.52) |
| t^2^ | -0.85 | * | (-1.65－ | -0.06) |  | -0.51 | |  | (-1.12－ | 0.11) |  | -0.63 | | * | (-1.18－ | -0.09) |
| t^3^ | 0.05 | * | (0.003－ | 0.09) |  | 0.03 | |  | (-0.004－ | 0.06) |  | 0.04 | |  | (0.007－ | 0.06) |
|  | **R^2^=0.4981** | |  |  |  | **R^2^=0.3712** | | |  |  |  | **R^2^=0.4441** | | |  |  |
| **30-day mortality** | | | | | | | | | | | | | | | | |
| Simple linear regression models with robust variance estimates | | | | | |  | | |  |  |  |  | | |  |  |
| Intercept | 92.52 | *** | (90.33－ | 94.71) |  | 93.08 | | *** | (90.92－ | 95.24) |  | 91.56 | | *** | (89.18－ | 93.94) |
| t | -0.53 | *** | (-0.76－ | -0.30) |  | -0.55 | | *** | (-0.76－ | -0.33) |  | -0.51 | | ** | (-0.77－ | -0.24) |
|  | R^2^=0.4774 | |  |  |  | R^2^=0.4960 | | |  |  |  | R^2^=0.4296 | | |  |  |
| Polynomial models with the quadratic term of “t” and with robust variance estimates | | | | | | | | | | |  |  | | |  |  |
| Intercept | 92.92 | *** | (90.14－ | 95.70) |  | 93.34 | *** | | (90.88－ | 95.80) |  | 92.12 | *** | | (88.72－ | 95.51) |
| t | -0.75 |  | (-2.22－ | 0.72) |  | -0.69 |  | | (-2.11－ | 0.72) |  | -0.81 |  | | (-2.41－ | 0.78) |
| t^2^ | 0.02 |  | (-0.10－ | 0.14) |  | 0.01 |  | | (-0.11－ | 0.13) |  | 0.03 |  | | (-0.10－ | 0.15) |
|  | R^2^=0.4835 | |  |  |  | R^2^=0.4986 | | |  |  |  | R^2^=0.4414 | | |  |  |
| **Polynomial models with the *quadratic* term and the *cubic* term of “t” and with robust variance estimates** | | | | | | | | | | |  |  | | |  |  |
| Intercept | 90.91 | *** | (89.49－ | 92.33) |  | 91.49 | | *** | (90.00－ | 92.97) |  | 89.83 | | *** | (88.37－ | 91.29) |
| t | 1.77 |  | (-0.89－ | 4.44) |  | 1.64 | |  | (-1.15－ | 4.44) |  | 2.06 | |  | (-0.53－ | 4.65) |
| t^2^ | -0.53 | * | (-1.06－ | -0.003) |  | -0.49 | |  | (-1.05－ | 0.06) |  | -0.60 | | * | (-1.10－ | -0.09) |
| t^3^ | 0.03 | * | (0.003－ | 0.06) |  | 0.03 | |  | (-0.001－ | 0.06) |  | 0.03 | | * | (0.008－ | 0.06) |
|  | **R^2^=0.6591** | |  |  |  | **R^2^=0.6455** | | |  |  |  | **R^2^=0.6679** | | |  |  |
| **180-day mortality** | | | | | | | | | | | | | | | | |
| Simple linear regression models with robust variance estimates | | | | | |  | | |  |  |  |  | | |  |  |
| Intercept | 94.09 | *** | (92.00－ | 96.19) |  | 94.46 | | *** | (92.43－ | 96.50) |  | 93.43 | | *** | (91.20－ | 95.66) |
| t | -0.59 | *** | (-0.82－ | -0.37) |  | -0.59 | | *** | (-0.81－ | -0.37) |  | -0.59 | | *** | (-0.85－ | -0.34) |
|  | R^2^=0.5276 | |  |  |  | R^2^=0.5403 | | |  |  |  | R^2^=0.5013 | | |  |  |
| Polynomial models with the quadratic term of “t” and with robust variance estimates | | | | | | | | | | |  |  | | |  |  |
| Intercept | 94.71 | *** | (92.07－ | 97.35) |  | 95.02 | *** | | (92.59－ | 97.44) |  | 94.23 | *** | | (91.05－ | 97.40) |
| t | -0.93 |  | (-2.36－ | 0.51) |  | -0.90 |  | | (-2.28－ | 0.49) |  | -1.03 |  | | (-2.58－ | 0.53) |
| t^2^ | 0.03 |  | (-0.09－ | 0.15) |  | 0.03 |  | | (-0.09－ | 0.14) |  | 0.04 |  | | (-0.09－ | 0.16) |
|  | R^2^=0.5405 | |  |  |  | R^2^=0.5512 | | |  |  |  | R^2^=0.5219 | | |  |  |
| **Polynomial models with the *quadratic* term and the *cubic* term of “t” and with robust variance estimates** | | | | | | | | | | |  |  | | |  |  |
| Intercept | 92.73 | *** | (91.28－ | 94.18) |  | 93.16 | | *** | (91.69－ | 94.63) |  | 91.98 | | *** | (90.56－ | 93.40) |
| t | 1.56 |  | (-1.14－ | 4.25) |  | 1.45 | |  | (-1.27－ | 4.16) |  | 1.80 | |  | (-1.78－ | 4.38) |
| t^2^ | -0.51 |  | (-1.04－ | 0.02) |  | -0.48 | |  | (-1.02－ | 0.06) |  | -0.58 | | * | (-1.08－ | -0.07) |
| t^3^ | 0.03 | * | (0.002－ | 0.06) |  | 0.03 | | * | (0.0001－ | 0.06) |  | 0.03 | | * | (0.008－ | 0.06) |
|  | **R^2^=0.6931** | |  |  |  | **R^2^=0.6898** | | |  |  |  | **R^2^=0.7099** | | |  |  |

^*^ p<0.05; ^**^p<0.01; ^***^p<0.001.

Abbreviations: CI, confidence interval; OHCA, out-of-hospital cardiac arrest.

^a^For the year 2000, t=0; t=1 for the year 2001, t=2 for the year 2002, and so on.
