# Supplementary material for: The Secular Trends in the Incidence Rate and Outcomes of Out-of-Hospital Cardiac Arrest in Taiwan—A Nationwide Population-Based Study
Source: PLoS One. 2015 Apr 15;10(4):e0122675. doi: 10.1371/journal.pone.0122675 (PMC4398054; doi:10.1371/journal.pone.0122675)
Supplement: S8 Table — (DOC) [file pone.0122675.s015.doc]

**S8 Table. Linear and polynomial regression models of annual mortality rates (%) among OHCA patients, for national data of Taiwan from 2000 to 2012, by age.**

|  | Aged 18~64 | | | | |  | Aged 65~74 | | | | |  | Aged 75~84 | | | | |  | | Aged 85+ | | | | |
| --- | --- | --- | --- | --- | --- | --- | --- | --- | --- | --- | --- | --- | --- | --- | --- | --- | --- | --- | --- | --- | --- | --- | --- | --- |
|  | Coefficient | | | 95%CI | |  | Coefficient | | | 95%CI | |  | Coefficient | | 95%CI | | |  | | Coefficient | | 95%CI | | |
| **1-day mortality** | | | | | | | | | | | | | | | | | | | | | | | | |
| Simple linear regression models with robust variance estimates | | | | | | | | | | | | | | | | | |  |  | |  |  |  |  |
| Intercept | 80.25 | *** | (77.53－ | | 82.97) |  | 85.08 | *** | (82.52－ | | 87.64) |  | 86.01 | *** | | (83.69－ | 88.33) |  | 80.60 | | *** | (77.10－ | 84.09) |  |
| t | -0.11 |  | (-0.36－ | | 0.13) |  | -0.61 | ** | (-0.90－ | | -0.32) |  | -0.41 | ** | | (-0.66－ | -0.17) |  | 0.26 | |  | (-0.11－ | 0.63) |  |
|  | R^2^=0.0297 | |  | |  |  | R^2^=0.4212 | |  | |  |  | R^2^=0.4040 | | |  |  |  | R^2^=0.1333 | | |  |  |  |
| Polynomial models with the quadratic term of “t” and with robust variance estimates | | | | | | | | | | | | | | | | | |  |  | |  |  |  |  |
| Intercept | 79.87 | *** | (77.56－ | | 82.17) |  | 85.65 | *** | (83.08－ | | 88.22) |  | 85.98 | *** | | (82.75－ | 89.21) |  | 78.86 | | *** | (76.06－ | 81.65) |  |
| t | 0.10 |  | (-1.40－ | | 1.60) |  | -0.92 |  | (-2.66－ | | 0.83) |  | -0.40 |  | | (-1.85－ | 1.06) |  | 1.21 | |  | (-0.34－ | 2.76) |  |
| t^2^ | -0.02 |  | (-0.15－ | | 0.11) |  | 0.03 |  | (-0.13－ | | 0.18) |  | -0.001 |  | | (-0.12－ | 0.12) |  | -0.08 | |  | (-0.21－ | 0.06) |  |
|  | R^2^=0.0375 | |  | |  |  | R^2^=0.4296 | |  | |  |  | R^2^=0.0.4040 | | |  |  |  | R^2^=0.2659 | | |  |  |  |
| **Polynomial models with the *quadratic* term and the *cubic* term of “t” and with robust variance estimates** | | | | | | | | | | | | | | | | | |  |  | |  |  |  |  |
| Intercept | 78.13 | *** | (76.16－ | | 80.09) |  | 83.11 | *** | (80.18－ | | 86.03) |  | 84.16 | *** | | (82.33－ | 85.99) |  | 76.11 | | *** | (73.32－ | 78.90) |  |
| t | 2.29 |  | (-1.32－ | | 5.89) |  | 2.28 |  | (-1.27－ | | 5.82) |  | 1.89 |  | | (-0.43－ | 4.21) |  | 4.67 | | ** | (1.89－ | 7.45) |  |
| t^2^ | -0.49 |  | (-1.21－ | | 0.22) |  | -0.67 |  | (-1.34－ | | 0.007) |  | -0.50 | * | | (-0.96－ | -0.04) |  | -0.83 | | ** | (-1.35－ | -0.31) |  |
| t^3^ | 0.03 |  | (-0.01－ | | 0.06) |  | 0.04 | * | (0.004－ | | 0.07) |  | 0.03 | * | | (0.004－ | 0.05) |  | 0.04 | | ** | (0.02－ | 0.07) |  |
|  | **R^2^=0.2233** | |  | |  |  | **R^2^=0.6218** | |  | |  |  | **R^2^=0.6071** | | |  |  |  | **R^2^=0.6433** | | |  |  |  |
| **30-day mortality** | | | | | | | | | | | | | | | | | | | | | | | | |
| Simple linear regression models with robust variance estimates | | | | | | | | | | | | | | | | | |  |  | |  |  |  |  |
| Intercept | 91.87 | *** | (89.64－ | | 94.10) |  | 94.10 | *** | (91.79－ | | 96.40) |  | 93.69 | *** | | (91.20－ | 96.18) |  | 88.46 | | *** | (84.81－ | 92.12) |  |
| t | -0.55 | *** | (-0.77－ | | -0.33) |  | -0.74 | *** | (-1.03－ | | -0.45) |  | -0.59 | ** | | (-0.86－ | -0.31) |  | 0.03 | |  | (-0.35－ | 0.41) |  |
|  | R^2^=0.4797 | |  | |  |  | R^2^=0.5695 | |  | |  |  | R^2^=0.5183 | | |  |  |  | R^2^=0.0021 | | |  |  |  |
| Polynomial models with the quadratic term of “t” and with robust variance estimates | | | | | | | | | | | | | | | | | |  |  | |  |  |  |  |
| Intercept | 92.31 | *** | (89.76－ | | 94.86) |  | 95.07 | *** | (92.10－ | | 98.34) |  | 94.03 | *** | | (90.17－ | 97.90) |  | 86.82 | | *** | (83.57－ | 90.07) |  |
| t | -0.78 |  | (-2.18－ | | 0.61) |  | -1.27 |  | (-2.90－ | | 0.36) |  | -0.77 |  | | (-2.46－ | 0.91) |  | 0.93 | |  | (-0.66－ | 2.52) |  |
| t^2^ | 0.02 |  | (-0.10－ | | 0.14) |  | 0.04 |  | (-0.10－ | | 0.18) |  | 0.02 |  | | (-0.12－ | 0.15) |  | -0.07 | |  | (-0.21－ | 0.06) |  |
|  | R^2^=0.4865 | |  | |  |  | R^2^=0.5919 | |  | |  |  | R^2^=0.5224 | | |  |  |  | R^2^=0.1397 | | |  |  |  |
| **Polynomial models with the *quadratic* term and the *cubic* term of “t” and with robust variance estimates** | | | | | | | | | | | | | | | | | |  |  | |  |  |  |  |
| Intercept | 90.79 | *** | (89.09－ | | 92.49) |  | 92.51 | *** | (90.30－ | | 94.72) |  | 91.83 | *** | | (89.94－ | 93.71) |  | 84.05 | | *** | (81.74－ | 86.37) |  |
| t | 1.12 |  | (-2.00－ | | 4.24) |  | 1.94 |  | (-0.88－ | | 4.77) |  | 2.00 |  | | (-0.44－ | 4.44) |  | 4.40 | | ** | (1.81－ | 6.99) |  |
| t^2^ | -0.39 |  | (-1.02－ | | 0.24) |  | -0.65 | * | (-1.19－ | | -0.12) |  | -0.59 | * | | (-1.06－ | -0.11) |  | -0.83 | | ** | (-1.32－ | -0.33) |  |
| t^3^ | 0.02 |  | (0.01－ | | 0.06) |  | 0.04 | * | (0.01－ | | 0.07) |  | 0.03 | * | | (0.008－ | 0.06) |  | 0.04 | | ** | (0.02－ | 0.07) |  |
|  | **R^2^=0.5819** | |  | |  |  | **R^2^=0.7683** | |  | |  |  | **R^2^=0.7137** | | |  |  |  | **R^2^=0.5840** | | |  |  |  |
| **180-day mortality** | | | | | | | | | | | | | | | | | | | | | | | | |
| Simple linear regression models with robust variance estimates | | | | | | | | | | | | | | | | | |  |  | |  |  |  |  |
| Intercept | 93.24 | *** | (91.09－ | | 95.39) |  | 95.75 | *** | (93.62－ | | 97.89) |  | 95.46 | *** | | (93.13－ | 97.80) |  | 90.02 | | *** | (86.51－ | 93.53) |  |
| t | -0.60 | *** | (-0.83－ | | -0.36) |  | -0.80 | *** | (-1.08－ | | -0.52) |  | -0.66 | *** | | (-0.92－ | -0.40) |  | -0.03 | |  | (-0.39－ | 0.33) |  |
|  | R^2^=0.5207 | |  | |  |  | R^2^=0.6239 | |  | |  |  | R^2^=0.5613 | | |  |  |  | R^2^=0.0018 | | |  |  |  |
| Polynomial models with the quadratic term of “t” and with robust variance estimates | | | | | | | | | | | | | | | | | |  |  | |  |  |  |  |
| Intercept | 94.03 | *** | (91.36－ | | 96.71) |  | 96.93 | *** | (94.33－ | | 99.53) |  | 96.12 | *** | | (92.57－ | 99.67) |  | 88.44 | | *** | (85.46－ | 91.42) |  |
| t | -1.03 |  | (-2.44－ | | 0.39) |  | -1.44 |  | (-2.93－ | | 0.06) |  | -1.02 |  | | (-2.66－ | 0.63) |  | 0.83 | |  | (-0.69－ | 2.36) |  |
| t^2^ | 0.04 |  | (-0.08－ | | 0.15) |  | 0.05 |  | (-0.07－ | | 0.18) |  | 0.03 |  | | (-0.10－ | 0.16) |  | -0.07 | |  | (-0.20－ | 0.06) |  |
|  | R^2^=0.5417 | |  | |  |  | R^2^=0.6546 | |  | |  |  | R^2^=0.5739 | | |  |  |  | R^2^=0.1340 | | |  |  |  |
| **Polynomial models with the *quadratic* term and the *cubic* term of “t” and with robust variance estimates** | | | | | | | | | | | | | | | | | |  |  | |  |  |  |  |
| Intercept | 92.42 | *** | (90.77－ | | 94.07) |  | 94.59 | *** | (92.21－ | | 96.97) |  | 93.89 | *** | | (92.33－ | 95.45) |  | 85.82 | | *** | (83.24－ | 88.40) |  |
| t | 1.00 |  | (-2.01－ | | 4.01) |  | 1.49 |  | (-1.27－ | | 4.26) |  | 1.79 |  | | (-0.76－ | 4.33) |  | 4.13 | | ** | (1.43－ | 6.83) |  |
| t^2^ | -0.40 |  | (-1.01－ | | 0.21) |  | -0.58 | * | (-1.11－ | | -0.06) |  | -0.58 | * | | (-1.08－ | -0.08) |  | -0.79 | | ** | (-1.30－ | -0.27) |  |
| t^3^ | 0.02 |  | (-0.008－ | | 0.06) |  | 0.04 | * | (0.008－ | | 0.06) |  | 0.03 | * | | (0.008－ | 0.06) |  | 0.04 | | ** | (0.01－ | 0.07) |  |
|  | **R^2^=0.6406** | |  | |  |  | **R^2^=0.7929** | |  | |  |  | **R^2^=0.7405** | | |  |  |  | **R^2^=0.5491** | | |  |  |  |

^*^ p<0.05; ^**^p<0.01; ^***^p<0.001.

Abbreviations: CI, confidence interval; OHCA, out-of-hospital cardiac arrest.

^a^For the year 2000, t=0; t=1 for the year 2001, t=2 for the year 2002, and so on.
