# Supplementary material for: The Secular Trends in the Incidence Rate and Outcomes of Out-of-Hospital Cardiac Arrest in Taiwan—A Nationwide Population-Based Study
Source: PLoS One. 2015 Apr 15;10(4):e0122675. doi: 10.1371/journal.pone.0122675 (PMC4398054; doi:10.1371/journal.pone.0122675)
Supplement: S9 Table — (DOC) [file pone.0122675.s016.doc]

**S9 Table. Linear and polynomial regression specifications that model the annual mortality rates (%) among OHCA patients in terms of a linear combination of the time period (t) as well as an autoregressive (AR) disturbance process, for national data of Taiwan from 2000 to 2012, by gender.**

|  | Both genders | | | |  | Men | | | | |  | Women | | | | |
| --- | --- | --- | --- | --- | --- | --- | --- | --- | --- | --- | --- | --- | --- | --- | --- | --- |
|  | Coefficient | | 95% CI | |  | Coefficient | | | 95% CI | |  | Coefficient | | | 95% CI | |
| **1-day mortality** | | | | | | | | | | | | | | | | |
| Simple linear specifications with robust variance estimates | | | | | |  | | |  |  |  |  | | |  |  |
| Intercept | 85.25 | *** | (79.33－ | 91.18) |  | 83.74 | | *** | (80.81－ | 86.68) |  | 81.25 | | *** | (78.49－ | 84.02) |
| t | -0.50 |  | (-1.28－ | 0.27) |  | -0.26 | |  | (-0.60－ | 0.08) |  | -0.19 | |  | (-0.50－ | 0.11) |
| Lag of 1 | -0.06 |  | (-0.82－ | 0.71) |  | 0.45 | | ** | (0.12－ | 0.77) |  | 0.71 | | *** | (0.46－ | 0.96) |
| Lag of 2 | -0.12 |  | (-1.26－ | 1.01) |  | -0.49 | | *** | (-0.75－ | -0.23) |  | -0.60 | | ** | (-0.98－ | -0.21) |
| Lag of 3 | -0.01 |  | (-0.73－ | 0.71) |  | 0.02 | |  | (-0.28－ | 0.32) |  | 0.10 | |  | (-0.19－ | 0.39) |
| Sigma^b^ | 3.16 | *** | (2.09－ | 4.23) |  | 1.79 | | ** | (0.64－ | 2.94) |  | 1.66 | | ** | (0.64－ | 2.68) |
| Polynomial specifications with the quadratic term of “t” and with robust variance estimates | | | | | | | | | | |  |  | | |  |  |
| Intercept | 85.72 | *** | (73.17－ | 98.28) |  | 83.31 | *** | | (80.70－ | 85.93) |  | 81.17 | *** | | (78.28－ | 84.06) |
| t | -0.72 |  | (-4.72－ | 3.29) |  | -0.04 |  | | (-0.92－ | 0.85) |  | -0.15 |  | | (-1.30－ | 1.00) |
| t^2^ | 0.02 |  | (-0.25－ | 0.28) |  | -0.02 |  | | (-0.10－ | 0.06) |  | -0.004 |  | | (-0.10－ | 0.09) |
| Lag of 1 | -0.08 |  | (-1.15－ | 0.99) |  | 0.45 | *** | | (0.20－ | 0.69) |  | 0.72 | *** | | (0.43－ | 1.00) |
| Lag of 2 | -0.16 |  | (-1.87－ | 1.56) |  | -0.50 | ** | | (-0.77－ | -0.22) |  | -0.60 | ** | | (-0.98－ | -0.22) |
| Lag of 3 | -0.001 |  | (-0.76－ | 0.76) |  | 0.02 |  | | (-0.25－ | 0.29) |  | 0.10 |  | | (-0.19－ | 0.40) |
| Sigma^b^ | 3.15 | *** | (2.11－ | 4.18) |  | 1.78 | ** | | (0.67－ | 2.89) |  | 1.66 | ** | | (0.65－ | 2.66) |
| **Polynomial specifications with the *quadratic* term and the *cubic* term of “t” and with robust variance estimates** | | | | | | | | | | |  |  | | |  |  |
| Intercept | 82.68 | *** | (77.86－ | 87.49) |  | 79.80 | | *** | (77.84－ | 81.75) |  | 78.20 | | *** | (76.12－ | 80.28) |
| t | 3.21 | * | (0.39－ | 6.02) |  | 3.79 | | *** | (2.22－ | 5.37) |  | 3.60 | | ** | (1.38－ | 5.82) |
| t^2^ | -0.86 | *** | (-1.33－ | -0.39) |  | -0.83 | | *** | (-1.15－ | -0.51) |  | -0.83 | | ** | (-1.30－ | -0.35) |
| t^3^ | 0.05 | *** | (0.03－ | 0.07) |  | 0.05 | | *** | (0.03－ | 0.06) |  | 0.05 | | ** | (0.02－ | 0.07) |
| Lag of 1 | -0.62 |  | (-1.33－ | 0.09) |  | -0.19 | |  | (-0.80－ | 0.42) |  | 0.05 | |  | (-0.84－ | 0.94) |
| Lag of 2 | -0.47 |  | (-1.31－ | 0.37) |  | -0.73 | | *** | (-0.94－ | -0.51) |  | -0.69 | | *** | (-0.92－ | -0.45) |
| Lag of 3 | -0.19 |  | (-0.89－ | 0.51) |  | -0.42 | |  | (-0.93－ | 0.08) |  | -0.24 | |  | (-1.00－ | 0.52) |
| Sigma^b^ | 2.19 | *** | (1.81－ | 2.56) |  | 1.03 | | *** | (0.72－ | 1.34) |  | 1.07 | | *** | (0.74－ | 1.40) |

**S9 Table. (Continued).**

|  | Both genders | | | |  | Men | | | | |  | Women | | | | |
| --- | --- | --- | --- | --- | --- | --- | --- | --- | --- | --- | --- | --- | --- | --- | --- | --- |
|  | Coefficient | | 95% CI | |  | Coefficient | | | 95% CI | |  | Coefficient | | | 95% CI | |
| **30-day mortality** | | | | | | | | | | | | | | | | |
| Simple linear specifications with robust variance estimates | | | | | |  | | |  |  |  |  | | |  |  |
| Intercept | 92.50 | *** | (90.11－ | 94.89) |  | 93.17 | | *** | (90.71－ | 95.64) |  | 91.38 | | *** | (88.94－ | 93.83) |
| t | -0.54 | *** | (-0.81－ | -0.26) |  | -0.57 | | *** | (-0.86－ | -0.28) |  | -0.49 | | ** | (-0.78－ | -0.20) |
| Lag of 1 | 0.55 | *** | (0.31－ | 0.79) |  | 0.46 | | ** | (0.20－ | 0.72) |  | 0.64 | | ** | (0.21－ | 1.07) |
| Lag of 2 | -0.45 | * | (-0.87－ | -0.04) |  | -0.42 | | * | (-0.78－ | -0.06) |  | -0.50 | |  | (-1.04－ | 0.05) |
| Lag of 3 | -0.0002 |  | (-0.27－ | 0.27) |  | -0.06 | |  | (-0.34－ | 0.23) |  | 0.08 | |  | (-0.34－ | 0.49) |
| Sigma^b^ | 1.68 | ** | (0.65－ | 2.72) |  | 1.69 | | ** | (0.57－ | 2.82) |  | 1.74 | | *** | (0.88－ | 2.60) |
| Polynomial specifications with the quadratic term of “t” and with robust variance estimates | | | | | | | | | | |  |  | | |  |  |
| Intercept | 92.86 | *** | (89.90－ | 95.83) |  | 93.45 | *** | | (90.68－ | 96.22) |  | 91.70 | *** | | (87.97－ | 95.43) |
| t | -0.72 |  | (-1.79－ | 0.35) |  | -0.71 |  | | (-1.61－ | 0.20) |  | -0.65 |  | | (-2.14－ | 0.84) |
| t^2^ | 0.01 |  | (-0.07－ | 0.10) |  | 0.01 |  | | (-0.06－ | 0.09) |  | 0.01 |  | | (-0.10－ | 0.12) |
| Lag of 1 | 0.53 | *** | (0.31－ | 0.75) |  | 0.45 | *** | | (0.18－ | 0.71) |  | 0.62 | * | | (0.14－ | 1.11) |
| Lag of 2 | -0.45 | * | (-0.82－ | -0.08) |  | -0.42 | * | | (-0.75－ | 0.09) |  | -0.49 |  | | (-0.99－ | 0.01) |
| Lag of 3 | -0.01 |  | (-0.27－ | 0.25) |  | -0.06 |  | | (-0.36－ | 0.23) |  | 0.07 |  | | (-0.37－ | 0.51) |
| Sigma^b^ | 1.68 | ** | (0.60－ | 2.75) |  | 1.69 | ** | | (0.54－ | 2.84) |  | 1.74 | *** | | (0.84－ | 2.64) |
| **Polynomial specifications with the *quadratic* term and the *cubic* term of “t” and with robust variance estimates** | | | | | | | | | | |  |  | | |  |  |
| Intercept | 90.09 | *** | (88.35－ | 91.83) |  | 90.38 | | *** | (88.43－ | 92.34) |  | 89.30 | | *** | (87.59－ | 91.00) |
| t | 2.70 | ** | (0.87－ | 4.52) |  | 2.80 | | ** | (1.04－ | 4.56) |  | 2.67 | | ** | (0.67－ | 4.66) |
| t^2^ | -0.74 | *** | (-1.14－ | -0.34) |  | -0.75 | | *** | (-1.12－ | -0.38) |  | -0.74 | | ** | (-1.17－ | -0.30) |
| t^3^ | 0.04 | *** | (0.02－ | 0.07) |  | 0.04 | | *** | (0.02－ | 0.06) |  | 0.04 | | ** | (0.02－ | 0.07) |
| Lag of 1 | -0.04 |  | (-0.73－ | 0.66) |  | -0.16 | |  | (-0.87－ | 0.54) |  | 0.13 | |  | (-0.38－ | 0.63) |
| Lag of 2 | -0.65 | *** | (-0.93－ | -0.36) |  | -0.64 | | *** | (-0.88－ | -0.39) |  | -0.66 | | ** | (-1.03－ | -0.28) |
| Lag of 3 | -0.31 |  | (-0.87－ | 0.26) |  | -0.45 | |  | (-0.99－ | 0.09) |  | -0.06 | |  | (-0.50－ | 0.38) |
| Sigma^b^ | 1.09 | *** | (0.69－ | 1.49) |  | 1.06 | | *** | (0.71－ | 1.42) |  | 1.19 | | *** | (0.71－ | 1.67) |

**S9 Table. (Continued).**

|  | Both genders | | | |  | Men | | | | |  | Women | | | | |
| --- | --- | --- | --- | --- | --- | --- | --- | --- | --- | --- | --- | --- | --- | --- | --- | --- |
|  | Coefficient | | 95% CI | |  | Coefficient | | | 95% CI | |  | Coefficient | | | 95% CI | |
| **180-day mortality** | | | | | | | | | | | | | | | | |
| Simple linear specifications with robust variance estimates | | | | | |  | | |  |  |  |  | | |  |  |
| Intercept | 94.08 | *** | (91.79－ | 96.37) |  | 94.49 | | *** | (92.19－ | 96.79) |  | 93.37 | | *** | (91.16－ | 95.59) |
| t | -0.60 | *** | (-0.86－ | -0.34) |  | -0.60 | | *** | (-0.88－ | -0.33) |  | -0.59 | | *** | (-0.85－ | -0.34) |
| Lag of 1 | 0.60 | ** | (0.25－ | 0.94) |  | 0.52 | | *** | (0.24－ | 0.80) |  | 0.67 | | ** | (0.23－ | 1.12) |
| Lag of 2 | -0.48 | * | (-0.92－ | -0.04) |  | -0.45 | | * | (-0.82－ | -0.08) |  | -0.49 | |  | (-1.06－ | 0.08) |
| Lag of 3 | 0.01 |  | (-0.29－ | 0.31) |  | -0.03 | |  | (-0.32－ | 0.27) |  | 0.03 | |  | (-0.36－ | 0.42) |
| Sigma^b^ | 1.64 | ** | (0.62－ | 2.67) |  | 1.64 | | ** | (0.58－ | 2.69) |  | 1.69 | | *** | (0.84－ | 2.55) |
| Polynomial specifications with the quadratic term of “t” and with robust variance estimates | | | | | | | | | | |  |  | | |  |  |
| Intercept | 94.67 | *** | (91.8－ | 97.5) |  | 95.07 | *** | | (92.34－ | 97.80) |  | 94.08 | *** | | (90.58－ | 97.57) |
| t | -0.89 |  | (-1.90－ | 0.12) |  | -0.89 |  | | (-1.79－ | 0.01) |  | -0.94 |  | | (-2.31－ | 0.42) |
| t^2^ | 0.02 |  | (-0.06－ | 0.10) |  | 0.02 |  | | (-0.05－ | 0.10) |  | 0.03 |  | | (-0.08－ | 0.13) |
| Lag of 1 | 0.56 | *** | (0.31－ | 0.81) |  | 0.49 | *** | | (0.26－ | 0.72) |  | 0.63 | ** | | (0.23－ | 1.03) |
| Lag of 2 | -0.47 | ** | (-0.83－ | -0.12) |  | -0.44 | ** | | (-0.74－ | -0.14) |  | -0.48 | * | | (-0.95－ | -0.01) |
| Lag of 3 | -0.01 |  | (-0.28－ | 0.26) |  | -0.05 |  | | (-0.33－ | 0.24) |  | 0.01 |  | | (-0.36－ | 0.38) |
| Sigma^b^ | 1.63 | ** | (0.54－ | 2.72) |  | 1.62 | ** | | (0.51－ | 2.74) |  | 1.68 | *** | | (0.74－ | 2.61) |
| **Polynomial specifications with the *quadratic* term and the *cubic* term of “t” and with robust variance estimates** | | | | | | | | | | |  |  | | |  |  |
| Intercept | 91.94 | *** | (90.13－ | 93.76) |  | 92.03 | | *** | (90.14－ | 93.92) |  | 91.68 | | *** | (90.11－ | 93.22) |
| t | 2.46 | * | (0.51－ | 4.41) |  | 2.61 | | ** | (0.93－ | 4.30) |  | 2.29 | | * | (0.21－ | 4.36) |
| t^2^ | -0.71 | ** | (-1.14－ | -0.29) |  | -0.74 | | *** | (-1.09－ | -0.38) |  | -0.70 | | ** | (-1.16－ | -0.23) |
| t^3^ | 0.04 | ** | (0.02－ | 0.07) |  | 0.04 | | *** | (0.02－ | 0.06) |  | 0.04 | | ** | (0.01－ | 0.07) |
| Lag of 1 | -0.02 |  | (-0.78－ | 0.74) |  | -0.16 | |  | (-0.87－ | 0.55) |  | 0.13 | |  | (-0.44－ | 0.71) |
| Lag of 2 | -0.64 | *** | (-0.90－ | -0.38) |  | -0.64 | | *** | (-0.87－ | -0.42) |  | -0.63 | | ** | (-0.99－ | -0.26) |
| Lag of 3 | -0.31 |  | (-0.91－ | 0.28) |  | -0.46 | |  | (-1.00－ | 0.07) |  | -0.13 | |  | (-0.65－ | 0.39) |
| Sigma^b^ | 1.09 | *** | (0.69－ | 1.49) |  | 0.99 | | *** | (0.65－ | 1.34) |  | 1.21 | | *** | (0.71－ | 1.70) |

^*^ p<0.05; ^**^p<0.01; ^***^p<0.001.

Abbreviations: CI, confidence interval; OHCA, out-of-hospital cardiac arrest.

^a^For the year 2000, t=0; t=1 for the year 2001, t=2 for the year 2002, and so on. The models include lags of 1, 2 and 3 of the structural disturbance.

^b^Sigma is ***the estimated standard deviation of the white-noise disturbance.***
