# Supplementary material for: The Secular Trends in the Incidence Rate and Outcomes of Out-of-Hospital Cardiac Arrest in Taiwan—A Nationwide Population-Based Study
Source: PLoS One. 2015 Apr 15;10(4):e0122675. doi: 10.1371/journal.pone.0122675 (PMC4398054; doi:10.1371/journal.pone.0122675)
Supplement: S10 Table — (DOC) [file pone.0122675.s017.doc]

**S10 Table. Linear and polynomial regression specifications that model the annual mortality rates (%) among OHCA patients in terms of a linear combination of the time period (t) as well as an autoregressive (AR) disturbance process, for national data of Taiwan from 2000 to 2012, by age.**

|  | Aged 18~64 | | | | |  | Aged 65~74 | | | | |  | Aged 75~84 | | | | |  | | Aged 85+ | | | | |
| --- | --- | --- | --- | --- | --- | --- | --- | --- | --- | --- | --- | --- | --- | --- | --- | --- | --- | --- | --- | --- | --- | --- | --- | --- |
|  | Coefficient | | | 95%CI | |  | Coefficient | | | 95%CI | |  | Coefficient | | 95%CI | | |  | | Coefficient | | 95%CI | | |
| **1-day mortality** | | | | | | | | | | | | | | | | | | | | | | | | |
| Simple linear specification with robust variance estimates | | | | | | | | | | | | | | | | | |  |  | |  |  |  |  |
| Intercept | 80.53 | *** | (77.59－ | | 83.48) |  | 85.04 | *** | (82.33－ | | 87.76) |  | 85.69 | *** | | (82.65－ | 88.72) |  | 80.63 | | *** | (75.96－ | 85.30) |  |
| t | -0.16 |  | (-0.50－ | | 0.19) |  | -0.60 | *** | (-0.93－ | | -0.28) |  | -0.37 |  | | (-0.76－ | 0.02) |  | 0.26 | |  | (-0.27－ | 0.79) |  |
| Lag of 1 | 0.23 |  | (-0.21－ | | 0.68) |  | 0.77 | *** | (0.59－ | | 0.96) |  | 0.56 |  | | (-0.15－ | 1.28) |  | 0.75 | | *** | (0.43－ | 1.06) |  |
| Lag of 2 | -0.36 | * | (-0.64－ | | -0.08) |  | -0.69 | *** | (-1.06－ | | -0.32) |  | -0.52 |  | | (-1.23－ | 0.18) |  | -0.50 | | * | (-0.92－ | -0.07) |  |
| Lag of 3 | -0.16 |  | (-0.54－ | | 0.23) |  | 0.17 |  | (-0.001－ | | 0.34) |  | 0.13 |  | | (-0.66－ | 0.92) |  | 0.04 | |  | (-0.56－ | 0.63) |  |
| Sigma^b^ | 2.05 | ** | (0.74－ | | 3.36) |  | 1.85 | *** | (0.82－ | | 2.88) |  | 1.57 | *** | | (0.96－ | 2.18) |  | 1.88 | | *** | (0.84－ | 2.92) |  |
| Polynomial specifications with the quadratic term of “t” and with robust variance estimates | | | | | | | | | | | | | | | | | |  |  | |  |  |  |  |
| Intercept | 80.12 | *** | (77.22－ | | 83.02) |  | 85.53 | *** | (82.83－ | | 88.23) |  | 85.35 | *** | | (81.23－ | 89.46) |  | 79.16 | | *** | (76.08－ | 82.23) |  |
| t | 0.05 |  | (-0.79－ | | 0.89) |  | -0.86 |  | (-1.97－ | | 0.25) |  | -0.20 |  | | (-1.65－ | 1.25) |  | 1.13 | | * | (0.04－ | 2.23) |  |
| t^2^ | -0.02 |  | (-0.09－ | | 0.05) |  | 0.02 |  | (-0.08－ | | 0.12) |  | -0.01 |  | | (-0.12－ | 0.09) |  | -0.07 | |  | (-0.16－ | 0.02) |  |
| Lag of 1 | 0.23 |  | (-0.15－ | | 0.62) |  | 0.76 | *** | (0.56－ | | 0.96) |  | 0.59 |  | | (-0.18－ | 1.35) |  | 0.65 | | *** | (0.46－ | 0.85) |  |
| Lag of 2 | -0.36 | * | (-0.66－ | | -0.07) |  | -0.68 | *** | (-1.02－ | | -0.34) |  | -0.54 |  | | (-1.28－ | 0.21) |  | -0.52 | | * | (-0.94－ | -0.10) |  |
| Lag of 3 | -0.15 |  | (-0.51－ | | 0.20) |  | 0.15 |  | (-0.03－ | | 0.34) |  | 0.15 |  | | (-0.71－ | 1.00) |  | 0.03 | |  | (-0.34－ | 0.41) |  |
| Sigma^b^ | 2.04 | ** | (0.74－ | | 3.33) |  | 1.84 | *** | (0.76－ | | 2.92) |  | 1.56 | ** | | (1.00－ | 2.12) |  | 1.77 | | *** | (0.78－ | 2.76) |  |
| **Polynomial specification with the *quadratic* term and the *cubic* term of “t” and with robust variance estimates** | | | | | | | | | | | | | | | | | |  |  | |  |  |  |  |
| Intercept | 76.32 | *** | (73.78－ | | 78.86) |  | 81.43 | *** | (78.74－ | | 84.11) |  | 82.99 | *** | | (81.15－ | 84.82) |  | 74.99 | | *** | (72.45－ | 77.54) |  |
| t | 3.99 | *** | (1.99－ | | 5.99) |  | 3.85 | ** | (1.57－ | | 6.14) |  | 2.97 | *** | | (1.62－ | 4.32) |  | 5.67 | | *** | (3.74－ | 7.60) |  |
| t^2^ | -0.85 | *** | (-1.25－ | | -0.44) |  | -0.99 | *** | (-1.45－ | | -0.53) |  | -0.73 | *** | | (-0.99－ | -0.46) |  | -1.03 | | *** | (-1.40－ | -0.65) |  |
| t^3^ | 0.05 | *** | (0.02－ | | 0.07) |  | 0.06 | *** | (0.03－ | | 0.08) |  | 0.04 | *** | | (0.03－ | 0.06) |  | 0.05 | | *** | (0.03－ | 0.07) |  |
| Lag of 1 | -0.29 |  | (-0.87－ | | 0.28) |  | -0.02 |  | (-0.89－ | | 0.86) |  | 0.05 |  | | (-0.24－ | 0.35) |  | -0.31 | |  | (-1.18－ | 0.56) |  |
| Lag of 2 | -0.67 | *** | (-0.95－ | | -0.38) |  | -0.73 | *** | (-0.91－ | | -0.55) |  | -0.76 | *** | | (-1.14－ | -0.38) |  | -0.69 | | *** | (-0.92－ | -0.46) |  |
| Lag of 3 | -0.51 | ** | (-0.94－ | | -0.09) |  | -0.33 |  | (-1.10－ | | 0.44) |  | -0.18 |  | | (-0.68－ | 0.32) |  | -0.41 | |  | (-1.21－ | 0.38) |  |
| Sigma^b^ | 1.30 | *** | (0.90－ | | 1.69) |  | 1.10 | *** | (0.75－ | | 1.46) |  | 0.89 | *** | | (0.63－ | 1.15) |  | 1.03 | | *** | (0.72－ | 1.33) |  |

**S10 Table. (Continued).**

|  | Aged 18~64 | | | | |  | Aged 65~74 | | | | |  | Aged 75~84 | | | | |  | | Aged 85+ | | | | |
| --- | --- | --- | --- | --- | --- | --- | --- | --- | --- | --- | --- | --- | --- | --- | --- | --- | --- | --- | --- | --- | --- | --- | --- | --- |
|  | Coefficient | | | 95%CI | |  | Coefficient | | | 95%CI | |  | Coefficient | | 95%CI | | |  | | Coefficient | | 95%CI | | |
| **30-day mortality** | | | | | | | | | | | | | | | | | | | | | | | | |
| Simple linear specification with robust variance estimates | | | | | | | | | | | | | | | | | |  |  | |  |  |  |  |
| Intercept | 92.03 | *** | (89.83－ | | 94.23) |  | 94.12 | *** | (91.58－ | | 96.65) |  | 93.43 | *** | | (90.72－ | 96.14) |  | 88.86 | | *** | (84.80－ | 92.92) |  |
| t | -0.58 | *** | (-0.84－ | | -0.32) |  | -0.74 | *** | (-1.10－ | | -0.37) |  | -0.56 | ** | | (-0.90－ | -0.21) |  | -0.02 | |  | (-0.50－ | 0.46) |  |
| Lag of 1 | 0.21 |  | (-0.13－ | | 0.54) |  | 0.74 | *** | (0.38－ | | 1.10) |  | 0.59 | ** | | (0.20－ | 0.99) |  | 0.65 | | *** | (0.34－ | 0.97) |  |
| Lag of 2 | -0.32 |  | (-0.66－ | | 0.02) |  | -0.53 |  | (-1.08－ | | 0.04) |  | -0.43 |  | | (-1.11－ | 0.24) |  | -0.38 | |  | (-0.82－ | 0.05) |  |
| Lag of 3 | -0.16 |  | (-0.56－ | | 0.24) |  | 0.01 |  | (-0.32－ | | 0.35) |  | 0.01 |  | | (-0.50－ | 0.53) |  | -0.17 | |  | (-0.75－ | 0.42) |  |
| Sigma^b^ | 1.88 | *** | (0.78－ | | 2.99) |  | 1.77 | *** | (0.88－ | | 2.66) |  | 1.75 | *** | | (1.02－ | 2.47) |  | 1.81 | | *** | (0.95－ | 2.68) |  |
| Polynomial specifications with the quadratic term of “t” and with robust variance estimates | | | | | | | | | | | | | | | | | |  |  | |  |  |  |  |
| Intercept | 92.45 | *** | (89.67－ | | 95.23) |  | 95.52 | *** | (91.90－ | | 99.13) |  | 93.52 | *** | | (88.78－ | 98.26) |  | 87.42 | | *** | (83.54－ | 91.30) |  |
| t | -0.79 |  | (-1.64－ | | 0.06) |  | -1.44 | * | (-2.71－ | | -0.17) |  | -0.60 |  | | (-2.43－ | 1.23) |  | 0.76 | |  | (-0.49－ | 2.02) |  |
| t^2^ | 0.02 |  | (-0.05－ | | 0.08) |  | 0.06 |  | (-0.05－ | | 0.16) |  | 0.003 |  | | (-0.13－ | 0.14) |  | -0.07 | |  | (-0.16－ | 0.03) |  |
| Lag of 1 | 0.18 |  | (-0.22－ | | 0.59) |  | 0.69 | *** | (0.48－ | | 0.90) |  | 0.58 | * | | (0.03－ | 1.14) |  | 0.59 | | *** | (0.37－ | 0.80) |  |
| Lag of 2 | -0.32 |  | (-0.64－ | | 0.001) |  | -0.52 | * | (-0.95－ | | -0.09) |  | -0.43 |  | | (-1.09－ | 0.23) |  | -0.38 | |  | (-0.86－ | 0.09) |  |
| Lag of 3 | -0.17 |  | (-0.58－ | | 0.24) |  | -0.03 |  | (-0.41－ | | 0.35) |  | 0.01 |  | | (-0.57－ | 0.59) |  | -0.17 | |  | (-0.58－ | 0.23) |  |
| Sigma^b^ | 1.87 | ** | (0.75－ | | 2.99) |  | 1.70 | *** | (0.70－ | | 2.69) |  | 1.75 | *** | | (1.01－ | 2.48) |  | 1.72 | | *** | (0.83－ | 2.61) |  |
| **Polynomial specification with the *quadratic* term and the *cubic* term of “t” and with robust variance estimates** | | | | | | | | | | | | | | | | | |  |  | |  |  |  |  |
| Intercept | 89.31 | *** | (87.00－ | | 91.63) |  | 91.91 | *** | (90.11－ | | 93.71) |  | 91.24 | *** | | (89.68－ | 92.80) |  | 83.80 | | *** | (81.98－ | 85.62) |  |
| t | 2.55 | ** | (0.70－ | | 4.39) |  | 2.80 | ** | (1.02－ | | 4.58) |  | 2.70 | ** | | (1.16－ | 4.24) |  | 4.91 | | *** | (3.07－ | 6.74) |  |
| t^2^ | -0.70 | *** | (-1.07－ | | -0.32) |  | -0.86 | *** | (-1.23－ | | -0.48) |  | -0.75 | *** | | (-1.07－ | -0.42) |  | -0.95 | | *** | (-1.35－ | -0.56) |  |
| t^3^ | 0.04 | *** | (0.02－ | | 0.06) |  | 0.05 | *** | (0.03－ | | 0.07) |  | 0.04 | *** | | (0.02－ | 0.06) |  | 0.05 | | *** | (0.03－ | 0.07) |  |
| Lag of 1 | -0.32 |  | (-0.93－ | | 0.30) |  | -0.02 |  | (-0.71－ | | 0.68) |  | 0.09 |  | | (-0.27－ | 0.45) |  | -0.16 | |  | (-0.83－ | 0.51) |  |
| Lag of 2 | -0.65 | *** | (-0.98－ | | -0.31) |  | -0.63 | *** | (-0.89－ | | -0.38) |  | -0.63 | ** | | (-1.08－ | -0.17) |  | -0.55 | | *** | (-0.84－ | -0.25) |  |
| Lag of 3 | -0.51 | * | (-0.95－ | | -0.06) |  | -0.40 |  | (-1.04－ | | 0.24) |  | -0.18 |  | | (-0.53－ | 0.17) |  | -0.44 | |  | (-1.08－ | 0.20) |  |
| Sigma^b^ | 1.26 | *** | (0.87－ | | 1.65) |  | 1.01 | *** | (0.74－ | | 1.28) |  | 1.16 | *** | | (0.79－ | 1.53) |  | 1.12 | | *** | (0.79－ | 1.44) |  |

**S10 Table. (Continued).**

|  | Aged 18~64 | | | | |  | Aged 65~74 | | | | |  | Aged 75~84 | | | | |  | | Aged 85+ | | | | |
| --- | --- | --- | --- | --- | --- | --- | --- | --- | --- | --- | --- | --- | --- | --- | --- | --- | --- | --- | --- | --- | --- | --- | --- | --- |
|  | Coefficient | | | 95%CI | |  | Coefficient | | | 95%CI | |  | Coefficient | | 95%CI | | |  | | Coefficient | | 95%CI | | |
| **180-day mortality** | | | | | | | | | | | | | | | | | | | | | | | | |
| Simple linear specification with robust variance estimates | | | | | | | | | | | | | | | | | |  |  | |  |  |  |  |
| Intercept | 93.33 | *** | (91.14－ | | 95.51) |  | 95.79 | *** | (93.63－ | | 97.95) |  | 95.29 | *** | | (92.95－ | 97.62) |  | 90.56 | | *** | (86.82－ | 94.29) |  |
| t | -0.62 | *** | (-0.89－ | | -0.36) |  | -0.79 | *** | (-1.12－ | | -0.47) |  | -0.64 | *** | | (-0.93－ | -0.35) |  | -0.10 | |  | (-0.54－ | 0.34) |  |
| Lag of 1 | 0.27 |  | (-0.03－ | | 0.56) |  | 0.86 | ** | (0.31－ | | 1.41) |  | 0.60 | *** | | (0.34－ | 0.87) |  | 0.64 | | *** | (0.33－ | 0.94) |  |
| Lag of 2 | -0.31 |  | (-0.67－ | | 0.05) |  | -0.64 | * | (-1.22－ | | -0.07) |  | -0.42 |  | | (-1.10－ | 0.25) |  | -0.38 | |  | (-0.81－ | 0.05) |  |
| Lag of 3 | -0.13 |  | (-0.56－ | | 0.29) |  | 0.09 |  | (-0.28－ | | 0.46) |  | 0.005 |  | | (-0.40－ | 0.41) |  | -0.20 | |  | (-0.79－ | 0.40) |  |
| Sigma^b^ | 1.89 | *** | (0.81－ | | 2.97) |  | 1.58 | *** | (0.91－ | | 2.24) |  | 1.77 | *** | | (0.91－ | 2.63) |  | 1.74 | | *** | (0.94－ | 2.54) |  |
| Polynomial specifications with the quadratic term of “t” and with robust variance estimates | | | | | | | | | | | | | | | | | |  |  | |  |  |  |  |
| Intercept | 94.15 | *** | (91.27－ | | 97.03) |  | 97.43 | *** | (94.18－ | | 100.68) |  | 95.85 | *** | | (91.76－ | 99.94) |  | 89.21 | | *** | (85.56－ | 92.87) |  |
| t | -1.03 | * | (-1.92－ | | -0.13) |  | -1.62 | ** | (-2.70－ | | -0.53) |  | -0.92 |  | | (-2.56－ | 0.72) |  | 0.62 | |  | (-0.53－ | 1.76) |  |
| t^2^ | 0.03 |  | (-0.03－ | | 0.10) |  | 0.07 |  | (-0.02－ | | 0.15) |  | 0.02 |  | | (-0.10－ | 0.14) |  | -0.06 | |  | (-0.14－ | 0.02) |  |
| Lag of 1 | 0.21 |  | (-0.20－ | | 0.61) |  | 0.77 | *** | (0.49－ | | 1.05) |  | 0.57 | ** | | (0.22－ | 0.92) |  | 0.57 | | *** | (0.36－ | 0.79) |  |
| Lag of 2 | -0.31 | * | (-0.62－ | | -0.006) |  | -0.61 | ** | (-0.97－ | | -0.26) |  | -0.42 |  | | (-1.03－ | 0.19) |  | -0.38 | |  | (-0.85－ | 0.08) |  |
| Lag of 3 | -0.16 |  | (-0.58－ | | 0.26) |  | 0.01 |  | (-0.33－ | | 0.35) |  | -0.01 |  | | (-0.43－ | 0.40) |  | -0.20 | |  | (-0.63－ | 0.23) |  |
| Sigma^b^ | 1.85 | ** | (0.73－ | | 2.97) |  | 1.48 | *** | (0.69－ | | 2.28) |  | 1.76 | *** | | (0.86－ | 2.66) |  | 1.66 | | *** | (0.82－ | 2.50) |  |
| **Polynomial specification with the *quadratic* term and the *cubic* term of “t” and with robust variance estimates** | | | | | | | | | | | | | | | | | |  |  | |  |  |  |  |
| Intercept | 90.98 | *** | (88.72－ | | 93.23) |  | 94.13 | *** | (92.55－ | | 95.72) |  | 93.44 | *** | | (92.05－ | 94.82) |  | 85.51 | | *** | (83.56－ | 87.46) |  |
| t | 2.39 | ** | (0.63－ | | 4.15) |  | 2.25 | ** | (0.59－ | | 3.90) |  | 2.42 | ** | | (0.74－ | 4.10) |  | 4.71 | | *** | (2.84－ | 6.57) |  |
| t^2^ | -0.70 | *** | (-1.06－ | | -0.34) |  | -0.76 | *** | (-1.12－ | | -0.41) |  | -0.73 | *** | | (-1.11－ | -0.36) |  | -0.93 | | *** | (-1.32－ | -0.53) |  |
| t^3^ | 0.04 | *** | (0.02－ | | 0.06) |  | 0.05 | *** | (0.03－ | | 0.07) |  | 0.04 | *** | | (0.02－ | 0.06) |  | 0.05 | | *** | (0.03－ | 0.07) |  |
| Lag of 1 | -0.33 |  | (-0.95－ | | 0.29) |  | 0.09 |  | (-0.57－ | | 0.75) |  | 0.04 |  | | (-0.46－ | 0.54) |  | -0.16 | |  | (-0.82－ | 0.50) |  |
| Lag of 2 | -0.64 | *** | (-0.96－ | | -0.33) |  | -0.67 | *** | (-0.91－ | | -0.43) |  | -0.60 | ** | | (-1.05－ | -0.15) |  | -0.54 | | *** | (-0.81－ | -0.27) |  |
| Lag of 3 | -0.53 | * | (-0.96－ | | -0.09) |  | -0.35 |  | (-0.99－ | | 0.29) |  | -0.23 |  | | (-0.62－ | 0.15) |  | -0.49 | |  | (-1.10－ | 0.12) |  |
| Sigma^b^ | 1.20 | *** | (0.81－ | | 1.59) |  | 0.90 | *** | (0.65－ | | 1.15) |  | 1.20 | *** | | (0.77－ | 1.64) |  | 1.08 | | *** | (0.75－ | 1.41) |  |

^*^ p<0.05; ^**^p<0.01; ^***^p<0.001.

Abbreviations: CI, confidence interval; OHCA, out-of-hospital cardiac arrest.

^a^For the year 2000, t=0; t=1 for the year 2001, t=2 for the year 2002, and so on. The models include lags of 1, 2 and 3 of the structural disturbance.

^b^Sigma is ***the estimated standard deviation of the white-noise disturbance.***
